# Coordination strategy and contract design of platform supply chain for large-scale sports events with low carbon preference

**DOI:** 10.1371/journal.pone.0311086

**Published:** 2024-12-02

**Authors:** Qianlan Chen, Siyi Mao, Ke Xu, Lin Lu

**Affiliations:** 1 School of Economics and Management, Guangxi Normal University, Guilin, China; 2 School of Management, José Rizal University, Mandaluyong City, Metro Manila, Philippine; Central Queensland University, AUSTRALIA

## Abstract

The organization of sports events, while generating economic benefits, inevitably imposes significant environmental pressures. Conducting green and low-carbon sports events have become a global consensus. In addressing the carbon emissions and benefit coordination issues on the production end of infrastructure construction for large-scale sports events, we considers the significant role of digital platforms in the industry’s low-carbon transformation and upgrade, and innovatively incorporates platforms as decision-making entities and investigates the equilibrium strategies for low-carbon cooperation under three different power structures: one led by the sports events materials supplier, one by the materials distributor, and one by the integrated service platform. Additionally, it designs related cost-sharing contracts. The findings suggest that: centralized decision-making is more conducive to aligning the interests of various entities within the sports events platform supply chain. Different power structures have distinct impacts on overall supply chain profits and carbon emissions. Specifically, the supply chain achieves the highest overall profit under the domination of the integrated service platform, while the lowest level of carbon reduction occurs under the domination of the materials distributor. These results provide strategic insights for the low-carbon development of sports events and the coordinated cooperation within platform supply chain.

## 1. Introduction

With the rapid development of the global economy and society, the sports industry has experienced vigorous growth. The hosting of sports events, particularly large-scale ones, not only brings significant economic benefits and social value to regions but also unavoidably results in corresponding carbon emissions due to the rigid consumption of energy sources [1,2]. Meanwhile, countries worldwide have long reached a consensus on the urgency of addressing the threat of climate change. In 2012, the United Nations Sustainable Development Conference centered around the "green economy" and sustainable development, emphasizing the need for nations to prioritize environmental energy issues. The "Transforming Our World: The 2030 Agenda for Sustainable Development" further underscores the vision of promoting human sustainable development. Against this backdrop, China has also put forward strategic goals for carbon peaking and carbon neutrality. While sports events may create environmental pressures, the universality of sports makes them a "messenger" for promoting sustainable development. By leveraging the promotional role of sports events, there is an opportunity to globally raise awareness of and address climate change issues and actively take measures. In 2015, the United Nations introduced the "Climate Neutrality" initiative, encouraging major sports events to adopt carbon offsetting for carbon neutrality. Five guidelines related to environmental protection and carbon reduction were proposed specifically for large-scale sports events [3].

According to statistics, carbon emissions generated during the preparatory phase of large-scale sports events, particularly from infrastructure construction, constitute the primary source of carbon emissions, accounting for 50% of the total carbon emissions from such events [4,5].Therefore, addressing the carbon emissions from infrastructure construction in the preparation process of sports events is a crucial step in achieving sustainable and low-carbon sports events.

The infrastructure construction for large-scale sports events typically involves the renovation or reconstruction of venues, a process that encompasses several critical supply chain stages, including the procurement of construction materials, transportation of goods, materials usage, and resource recycling. Supported by the internet and digital technologies, the integration of platforms has driven supply chain to evolve towards greater efficiency, intelligence, and transparency [6], and the sports events supply chain is no exception. In the context of the entire infrastructure construction process, the platform supply chain for large-scale sports events centers around an integrated service platform, bringing together key stakeholders such as sports events materials suppliers (sponsors), materials retailers, events contractors, and organizing committees. Through big data services, this platform facilitates the selection of high-quality collaboration partners for the various stakeholders, thereby accelerating the organization of large-scale sports events. Additionally, the integrated service platform possesses unique resources and advantages, providing the necessary technical support to all collaborating entities. Supply chain management reflects the decision-making behaviors of various stakeholders and process stages. Therefore, exploring low-carbon management of materials flows in large-scale sports events from a supply chain perspective—focusing on low-carbon practices throughout the preparation phase—enables the realization of full lifecycle management during the events organization stage.

The power structures within supply chain have long been a focal point of research in supply chain management [7]. The distribution of power affects the operational decisions and competitive relationships among the entities within the supply chain [8–10]. Due to their differing positions within the supply chain, the sources of benefits for each entity vary. Under different power structures, each entity makes decisions aimed at maximizing its own profit. In the context of the sports events platform supply chain, the roles of the integrated service platform, the materials supplier, and the materials distributor differ, and their positions are influenced by factors such as market resource control, technological dependence, and market scale.

Then, how should the balance between low-carbon objectives and profit be managed when the platform supply chain of sustainable sports events operates under different power structures? How should each entity make decisions? What are the differences in the impact of various power structures on carbon emissions, overall supply chain profit, and individual profits? Are there different contracts that can simultaneously enhance carbon reduction and overall supply chain profit? To effectively address these questions, we developed a game-theoretic model for the platform supply chain using the Stackelberg game framework. In this model, each entity within the platform supply chain has two options: to engage in low-carbon cooperation or to refrain from it. Under these two choices, we constructed both centralized and decentralized decision models for scenarios led by sports events materials supplier, integrated service platform, and materials distributor. Using backward induction, we solved for the optimal equilibrium solutions for each entity under different power structures to determine the coordination strategies for low-carbon cooperation. To enhance low-carbon cooperation among the parties, we also designed related cost-sharing contracts to achieve a more balanced profit distribution among the entities in the platform supply chain. Finally, numerical analysis was conducted to validate the effectiveness of the cost-sharing contracts and to explore their deeper properties. This provides concrete practical strategies and a research framework for the comprehensive and systematic sustainable development of sports events platform supply chain.

In previous research on supply chain power structures, scholars have predominantly focused on traditional supply chains, with limited consideration given to scenarios where platforms are included as decision-making entities. Thus, this study represents a significant advancement by incorporating platforms into the decision-making framework. Considering multiple decision-making scenarios can enrich game theory and cooperation theory, revealing the effectiveness of low-carbon cooperation and coordination mechanisms and the potential synergistic effects under different decision-making contexts. The contributions of this research are primarily reflected in the following aspects:

1. From the perspective of supply chain management, we investigate the sustainable development of sports events by constructing a platform supply chain consisting of a sports events materials supplier, a sports events materials distributor, an integrated service platform, and the events organizing committee. We explore full-chain low-carbon management based on sports events.
2. We break away from traditional supply chain research by focusing on platform supply chain and incorporating the integrated service platform into the power structure. We consider the coordination mechanisms for carbon reduction cooperation under the leadership of the integrated service platform.
3. We address various decision-making scenarios by separately examining carbon reduction cooperation strategies under centralized and decentralized decision-making within the platform supply chain. We also explore and compare the impacts of different power structures on the platform supply chain.
4. We design cost-sharing contracts for carbon reduction cooperation under three different power structures, coordinating the profits of all entities involved in carbon reduction efforts to incentivize active participation in carbon reduction cooperation within the sports events platform supply chain.

The remainder of this paper is organized as follows: Section 2 reviews the literature relevant to this study. Section 3 presents the research questions and model assumptions. Section 4 develops and solves equilibrium decision models under various scenarios and conducts a comparative analysis. Section 5 designs cost-sharing contracts. Section 6 performs a numerical analysis to explore deeper properties. Section 7 summarizes the study, provides related management recommendations, and discusses the limitations of the research and potential future directions.

## 2. Literature review

### 2.1. Sustainable sports events

Large-scale sports events, from the pre-events preparations to the events itself and the post-events closure, consume diverse resources, resulting in substantial carbon emissions [11]. In recent years, the excessive frequency of hosting sports events globally has led to an increasing emission of greenhouse gases, primarily attributed to the construction of events-related venues and transportation activities. This surge in emissions has exerted significant pressure on the environment [12,13], making the environmental sustainability of sports a prominent and pressing issue in the field of sports studies. The green development of sports events represents an inherently ecological developmental model. Former President of the International Olympic Committee: J. A. Samaranch, advocated for the incorporation of "environment" as the third dimension of the Olympic movement as early as the 1992 United Nations Conference on Environment and Development. In 1994, during the 12th Olympic Congress in Paris, the issues of sports and its environmental impact were first raised [14]. Olympic scholar Jean-Loup Chappelet (2008) further synthesized the experiences of various Winter Olympic Organizing Committees since 1970 in his work "Environmental Issues as an Olympic Legacy," elevating them into the concept of sustainable development in the Olympic movement [15]. Numerous scholars have scrutinized the impact of sports events on the environment and their interactive relationships. Governments typically implement environmental protection measures before and during large-scale sports events to maintain urban ecological balance and preserve renewable resources. These measures are often perceived as having a positive impact on environmental quality [16]. However, studies by Ma and Takeuchi (2020) and Zhang et al. (2016) on the impact of environmental emergency management measures on ecological improvement suggest that the "carry-over effect" of these measures leads to sustained short-term ecological improvement but lacks efficacy in the long term [17,18]. Zhang et al. (2016) also focused on the impact of the Youth Olympic Games on air pollution, discovering that carbon reduction measures implemented during the Games effectively curbed atmospheric pollution. However, post-Youth Olympics, there was a significant increase in air pollutants [19,20]. This indicates that governmental control measures during sports events can have a certain positive impact on environmental management but do not fundamentally address the root cause of carbon emissions from sports events.

In addition to the dual impacts of sports events on the environment, many scholars have explored pathways for achieving green and low-carbon sports events. Spanos et al. (2021), against the backdrop of the Fédération Internationale de Football Association (FIFA) World Cup 2022™, proposed a carbon-neutralization pathway by utilizing the carbon sequestration potential of trees planted in the Qatar Afforestation Project [21]. Ying Zhang (2023) approached the subject from a technological standpoint, employing AI technology to drive carbon-neutral management in sports events. The study suggested that a combination of the STIRPAT model, GRU neural networks, and transfer learning could accurately predict carbon emissions in sports events and effectively formulate carbon-neutralization strategies [22]. Carbon footprint has been a focal point in the research on low-carbon sports events. As a metric quantifying the environmental performance of sports events, organizations need to track and compare carbon footprint indicators to formulate environmental protection strategies. Carbon footprint typically refers to the global warming potential, essentially the unit of greenhouse gas conversion into a singular quantity of carbon dioxide [23,24]. In the context of sports events, most scholars have focused their research on travel, specifically transportation. Discussions have revolved around the measurement of carbon footprint, travel strategies, and related topics [25–28].

Existing research on carbon emissions and sustainable development in sports events has primarily focused on discussions surrounding values and significance. While some studies have touched upon strategies for achieving low-carbon sustainability in sports events, these efforts have been predominantly centered in the engineering domain. They lack managerial perspectives in terms of application mechanisms, and there is a noticeable scarcity of research exploring low-carbon sustainable development in sports events from the supply chain perspective.

### 2.2. Cooperative coordination mechanisms for carbon reduction in supply chain

Low-carbon supply chain management with a diverse range of research perspectives has consistently been a focal point in the field of supply chain research. Faced with the global challenge of climate change, pressures from governments, customers, and various stakeholders have compelled supply chain enterprises to integrate sustainability into supply chain management [29]. Consequently, low-carbon supply chains have emerged as a crucial means to address the demands of sustainable development. Exploring coordination mechanisms among low-carbon supply chains not only enables compliance with sustainability requirements but also facilitates efficiency improvements within supply chain enterprises. Many scholars have incorporated carbon constraints into the design of coordination strategies among supply chains. Lu et al. (2022) applied set pair analysis theory within the framework of carbon constraints to investigate the specific application of extended strategy generation methods in emergency cold chain logistics. They combined the context of the COVID-19 pandemic outbreak to design and assess various emergency plans for cold chain logistics under carbon constraint conditions [30]. Ding et al. (2016) considered environmental constraints and carbon regulations, studying the collaborative mechanisms between sustainable supply chains influenced by government environmental policy incentives [31]. Krishnendu Shaw et al. (2016), focusing on carbon emissions and carbon trading issues, utilized the Benders decomposition algorithm to study the design of a green supply chain network based on low-carbon opportunity constraints. The results indicated that carbon credit prices influence materials flows and costs within the supply chain network [32]. Cheng and Wang (2023) separately constructed carbon-constrained closed-loop supply chain network models under manufacturer-led and retailer-led modes. They explored the synergies between government policies and corporate operational measures. The research findings suggested that carbon quota allocation methods can promote profits between supply chains, and government incentives can enhance the sustainability level of the supply chain [33].

In the context of carbon emission reduction collaboration mechanisms, scholars have conducted research from various perspectives, including the cost sharing of carbon reduction, technological investments, and coordination strategies among entities at the optimal level of carbon reduction. The cost sharing of carbon emission reduction is mainly divided into cost sharing between manufacturers and retailers and the cost sharing of government subsidy policies under government regulations. Regarding the research on carbon emission reduction cost sharing between manufacturers and retailers, Liu and Li (2020) conducted a comparative analysis of the incentive effects on carbon reduction in the supply chain under cost-sharing contracts and two-part pricing contracts in the context of carbon trading policies [34]. Yu et al. (2020) considered reference emissions and cost learning effects, constructed a differential game model, analyzed equilibrium emission strategies and pricing strategies in cost-sharing contracts and revenue-sharing contracts, and pointed out that manufacturers are more inclined towards revenue-sharing contracts, while retailers prefer wholesale price contracts [35]. Zhou et al. (2016), taking into account the fairness psychology of retailers, analyzed the coordination issues of advertising contracts and cooperative advertising cost-sharing contracts between manufacturers and retailers affecting low-carbon supply chains [36]. Zhao et al. (2014) constructed three differential game models in a supply chain system with a single manufacturer and two retailers, finding that cost-sharing contracts can achieve Pareto improvement in manufacturer and retailer profits under certain conditions [37]. Ghosh and Shah (2015) studied two cost-sharing models—retailers providing cost-sharing contracts and retailers negotiating cost-sharing contracts with manufacturers, and analyzed the impact of these two cost-sharing contracts on decision-making [38].

In studies on government cost-sharing, many scholars, in conjunction with government policies, have explored the impact of carbon tax policies and subsidy policies on carbon emission reduction collaboration among upstream and downstream enterprises in the supply chain. Yi and Li (2018) found that subsidy policies can effectively promote energy-saving and emission reduction, while carbon tax policies may not necessarily be effective [39]. Similar conclusions were drawn by Drake et al. (2016), who observed that, compared to carbon tax policies, carbon cap-and-trade mechanisms can lead to greater profits for enterprises [40]. Yu (2022) also investigated the impact of carbon tax policies on two competitive emission reduction supply chains. In response to situations where collaboration among manufacturers in the two supply chains causes harm to retailers, relevant coordination contracts were designed [41]. Sinayi and Rasti-Barzoki (2018) considered manufacturers engaging in independent emission reduction research and development. Through the establishment of a game model, they studied supply chain decision-making problems under scenarios with and without government subsidies [42]. Cao et al. (2017) explored the impact of cap-and-trade policies and low-carbon subsidy policies on manufacturers’ production and carbon emission reduction levels. The results indicated that the level of carbon emission reduction increases with the increase in carbon trading prices but is unrelated to the unit low-carbon subsidy [43].

Regarding investment in carbon emission reduction technologies, Saberi et al. (2018) pointed out that investing in the application of carbon emission reduction technologies can reduce carbon emissions in the production processes of enterprises [44]. Building upon this, Wu and Li (2022) constructed a supply chain composed of manufacturers and retailers. They explored the impact of social responsibility models on carbon emission reduction and technological innovation decisions in a low-carbon supply chain. The study solved for the optimal decisions regarding supply chain carbon emission reduction intensity and technological innovation levels [45]. Liu et al. (2022) investigated the impact of the relationship between fresh food suppliers and retailers on the adoption of pre-cooling technology and the level of investment in carbon emission reduction research and development. By comparing the cooperative relationship between the two entities, they found that, in the case of collaboration between the two entities, the overall supply chain revenue and research and development investment levels for both technologies were higher compared to the non-collaborative scenario. In a decentralized situation, when the two entities do not collaborate, the overall supply chain revenue and research and development investment levels for both technologies depend on the relationship between retail and wholesale prices [46]. Chi et al. (2020) built a sustainable product-inventory model for carbon emission reduction technology collaborative investment under carbon total control and policies involving carbon trading and offset. They explored issues of competition and cooperation among enterprises, and the study indicated that collaborative investment in carbon emission reduction technology contributes to achieving carbon reduction goals and increasing overall supply chain profits [47]. Blockchain technology, as an emerging technology, has also been explored by many scholars in its integration into coordination mechanisms for carbon emission reduction in the supply chain. For example, Pan et al. (2023) focused on the collaborative emission reduction strategies between power generation companies and retailers under carbon tax policies. They constructed four decision models, including a Stackelberg game led by the power generation enterprise with simultaneous Nash bargaining decisions, vertical integration decisions of supply chain enterprises, cooperative carbon emission reduction game, and analyzed the changes in electricity prices, sustainability levels, power sales, and profits of supply chain members [48].

The existing research on collaborative mechanisms for carbon reduction in supply chain has already demonstrated considerable breadth and depth, with diverse perspectives being explored. This provides a strong foundation for the present study. Methodologically, the majority of scholars have employed game theory models to investigate the cooperative and coordination mechanisms among supply chain stakeholders in the context of carbon reduction. Game theory is particularly effective in uncovering the interactive dynamics among these stakeholders, allowing for the systematic modelling and analysis of the interdependencies between multiple participants. Within a supply chain, stakeholders often face conflicts and coordination challenges related to carbon reduction targets, resource allocation, and economic interests. Game theory offers a structured framework that enables scholars to analyze the changes in payoffs resulting from different strategic choices, thereby identifying the incentives and conditions necessary for cooperation. Furthermore, game theory assists researchers in assessing the feasibility of various cooperative models and the impact of policy interventions, thereby aiding decision-makers in formulating strategies that effectively promote carbon reduction. Therefore, we will also construct a three-party Stackelberg game model with different power structures to analyze the carbon reduction strategies of supply chain members, as well as the impact of consumer preferences for low-carbon products and platform technologies on carbon reduction and profitability.

### 2.3. Supply chain power structures

Existing research on supply chain power structures has achieved a certain breadth, with most scholars preferring to approach the topic from the perspectives of pricing decisions and coordination contracts. For example, Jiang et al. (2023) employed Stackelberg games, Nash games, and supply chain coordination methods to introduce manufacturer bankruptcy strategies into the prefabricated construction supply chain, investigating pricing, manufacturer bankruptcy, and supply chain coordination strategies under three different power structures [49]. Liu et al. (2023) explored the effects of anticipated regret and manufacturer fairness concerns on pricing and profits within a retailer-dominated supply chain, and proposed a revenue-sharing contract to optimize supply chain profits [50]. Xia et al. (2024) considered a two-tier supply chain consisting of a manufacturer and a retailer, examining the pricing decisions of upstream and downstream firms under retailer-led and manufacturer-led supply chains, as well as the impact of manufacturer equity holdings on signal transmission between upstream and downstream firms [51].

Certainly, many scholars have explored the impact of carbon emission reduction collaboration mechanisms from the perspectives of different power structures. For instance, Niu et al. (2022) examined the influence of power structures on the investment motivations of manufacturers in decarbonization technology under uncertain environments and the impact on retailer profits [52]. Huang et al. (2021) constructed a supply chain consisting of two manufacturers and a single retailer, studying the effects of power structures on carbon emission reduction decisions of supply chain enterprises under two different scenarios [53]. FAN et al. (2018) considered consumer preferences for low-carbon products and utilized game models to study the influence of different power structures on carbon emission reduction decisions of supply chain enterprises [54]. CHOI et al. (2013) focused on a three-tier closed-loop supply chain, investigating the impact of power structures on the closed-loop supply chain. They found that when retailers dominate, the closed-loop supply chain can achieve maximum profit [55]. Kou et al. (2022) constructed an analytical framework comprising a retailer and a manufacturer, exploring the value of cooperation under carbon cap-and-trade mechanisms from scenarios dominated by both entities. The study indicated that higher profits are achieved in the supply chain when the retailer dominates [56]. Gong et al. (2024) considered the behavioral trait of manufacturer overconfidence and developed a differential game model for a dual-channel green supply chain under three power structures: manufacturer-dominated, retailer-dominated, and a balanced power structure between manufacturer and retailer. The study explored the impact of manufacturer overconfidence and differences in power structures on the decisions and profits of supply chain members [57].

From the above researches, it is evident that the academic community has conducted extensive studies on power structures in low-carbon supply chain, but these studies mostly focus on general supply chain, with little consideration for scenarios where the core platform is one of the main entities in the supply chain. In practice, many platform companies, such as Alibaba, Amazon, have initiated carbon reduction collaborations with supply chain partners. However, in the academic field, there is scarce research on carbon reduction collaborations among platform supply chain. Therefore, we incorporate the platform into the supply chain power structure within the context of low-carbon sports events, exploring carbon reduction cooperation strategies in platform supply chain for sports events.

## 3. Problem description and basic assumptions

### 3.1. Problem description

Establishing cooperation agreements among supply chain enterprises can effectively integrate resources among them, achieving the maximization of each enterprise’s value. The advent of Internet information technology has brought about significant changes in the functions and roles of market participants [58]. In the context of sports events, the platform supply chain of sports events materials refers to the integration of various aspects of sports events-related materials procurement, supply, delivery, and management into a unified platform through digital technology and information means. This is done to realize a more efficient, transparent, and collaborative supply chain management system. In the context of sports events, the platform supply chain, relying on digital technology and the platform economy, plays a crucial role in carbon reduction efforts.

In the context of the global industrial value chain and carbon reduction efforts, the green transformation of the manufacturing industry is a crucial pathway to promoting economic sustainability [59]. Reducing carbon emissions from the production plays a crucial role in the sustainable development of sports events. This study focuses on the sustainability of sports events, specifically addressing the reduction of carbon emissions in the manufacturing process of sports events infrastructure. The research establishes a four-tier platform supply chain comprising a sports events materials supplier, a distributor, an integrated service platform, and an organizing committee. The materials supplier, with manufacturing capabilities, provide green building materials, green technology products, and green energy needed for sports events. Organizing committee, as a consumer, express a preference for low-carbon options. The integrated service platform offers transaction services and carbon reduction technical support, utilizing digital technology to assist suppliers in lowering carbon emissions. This approach ensures the overall sustainability of the sports events materials platform supply chain, without considering the use of sports events materials, the construction process, and materials resource recovery.

### 3.2. Basic assumptions

1. Assuming the initial carbon emissions per unit product provided by the sports events materials supplier is denoted as *e*_0_, the initial carbon emission cost is denoted as *te*_0_, where *t* represents the cost coefficient. In the process of carbon emission reduction collaboration among the various entities in the platform supply chain, the integrated service platform can provide carbon emission reduction technology support to sports events materials supplier enterprises with high emissions. Then assuming the integrated service platform provides a technological support intensity *h* per unit product, it will reduce the carbon emissions by *λe*_Δ_(*e*_0_>*e*_Δ_). Here, *h* represents the level of platform carbon emission reduction technology support input, and *λ* is the coefficient representing the impact or interference of platform technological support on the supplier’s carbon emission reduction, hereafter referred to as the coefficient of the impact of platform technological input on carbon reduction. Additionally, assuming that the carbon emission reduction level of sports events materials supplier products depends solely on the low-carbon collaboration technology input from the integrated service platform, the carbon emission cost for the materials supplier is denoted as *t*(*e*_0_>*e*_Δ_)).
2. Assuming the unit production cost of sports events materials supplier’s products is *c*:
$$c=c_{0}+t(e_{0})$$ (1)
  The unit production cost after the technological input from the integrated service platform is *c*_Δ_:
$$c_{\Delta }=c_{0}+t(e_{0}-e_{\Delta })$$ (2)
  Where *c*_0_ represents the initial manufacturing cost per unit product.
3. Assuming the order quantity of sports events materials distributor equals market demand, and market demand is a linear function of price and product carbon emissions.
$$D(p,e_{\Delta })=a-\alpha p-\beta (e_{0}-e_{\Delta })$$ (3)
p=w+b (4)
$$D(w,b,e_{\Delta })=a-\alpha (w+b)-\beta (e_{0}-e_{\Delta })$$ (5)
4. The integrated service platform, as a transaction intermediary, can provide various information services to sports events materials supplier and distributor. Therefore, it charges a uniform unit commission: m to both parties. Assuming that the sports events materials supplier, distributor, and integrated service platform are all rational decision-makers and make decisions based on complete information, each seeks the optimal strategy to maximize its own interests. The optimal profits for the sports events materials supplier, distributor, and integrated service platform can be represented by the following objective functions:

$$J_{M}=\max\;\pi_{M}=(w-c_{\Delta }-m)D(w,b,e_{\Delta })$$ (6)

$$J_{R}=\max\;\pi_{R}=(b-m)D(w,b,e_{\Delta })$$ (7)

$$J_{P}=\max\;\pi_{P}=2m\;D(w,b,e_{\Delta })-h^{2}\frac{u_{p}}{2}$$ (8)

The above is based on a scenario of decentralized decision-making. When the sports events materials supplier, distributor, and integrated service platform are in a centralized decision-making situation, all three parties aim to maximize the overall profit of the platform supply chain. The objective function in this case is:

$$J_{SC}=\max\;\pi_{SC}=(p-c_{\Delta })D(w,b,e_{\Delta })-h^{2}\frac{u_{p}}{2}$$ (9)

Where: Subscripts M, R, P, and SC represent the sports events materials supplier, sports events materials retailer, integrated service platform, and the overall platform supply chain, respectively. *b*>*m*>0, *u*_*p*_ denotes the cost coefficient for the level of low-carbon collaboration technology input by the integrated service platform, and *h*^2^*u*_*p*_/2 represents the cost required for platform technological input.

The specific variable parameters and symbols are shown in Table 1.

**Table 1:** Explanation of relevant parameters and symbols.

Parameters	Meaning	Range
t	Cost coefficient of per-unit product carbon emissions	t>0
h	Degree of carbon emission reduction technology support by the integrated service platform	h>0
e_0_	Initial carbon emission of the product	e_0_>0
e_Δ_	Carbon emission reduction amount per unit product empowered by platform technological support	e_0_>e_Δ_
λ	Platform technological support carbon emission reduction impact or interference coefficient	1>λ>0
c	Unit production cost of materials supplier’s product	c>0
c_0_	Initial unit product manufacturing cost	c_0_>0
c_Δ_	Post low-carbon collaboration, the unit product manufacturing cost of the materials supplier.	c_0_>c_Δ_>0
D	Market demand is a linear function concerning the price p and the product carbon reduction quantity e_Δ_.	D>0
w	Wholesale product pricing	p>w>c
p	Product retail price	p>w>c
b	Unit product market markup profit	b>m>0
a	Market capacity of the product	a>0
α	Price elasticity of demand	1>α>0
β	The sensitivity coefficient of demand to the carbon emissions of the product (consumer low-carbon preference level).	1>β>0
u_p_	Platform carbon reduction technology support cost coefficient	u_p_>0
m	The platform’s unit commission rate.	b>m>0
π_i_	Profit function	-
Subscripts M, R, P, SC	Representing the sports events materials supplier, distributor, integrated service platform, and the overall platform supply chain, respectively.	-

## 4. Model building and solution

### 4.1. Model analysis without low-carbon collaboration

#### 4.1.1. Decentralized decision-making

This study will explore the low-carbon collaboration coordination mechanisms when led by the sports events materials supplier, the sports events materials distributor, and the integrated service platform. Initially, an analysis is conducted on the optimal decisions when no low-carbon collaboration takes place among the various entities in the platform supply chain under sports events. This specifically refers to the decision-making when the integrated service platform does not provide carbon reduction technology support, serving as a baseline for subsequent research. The situations led by the sports events materials supplier, the distributor, and the integrated service platform are respectively denoted as MS, RS, and PS.

When the materials supplier takes a dominant position in the platform supply chain, the materials supplier first determines the wholesale price w then the distributor decides the retail price *p*, while simultaneously, the integrated service platform determines the platform commission rate *m*. Without considering carbon reduction collaboration, the market demand function at this point is *D*(*b*) = *a*−*α*(*w*+*b*)−*βe*_0_, where the product manufacturing cost is *c* = *c*_0_+*t*(*e*_0_).

Utilizing inverse inductive method to solve the optimal decisions of the materials supplier, materials distributor, and integrated service platform. Firstly, solving the optimal reactive decisions of the materials distributor and integrated service platform under the profit maximization condition. Given the unit commission rate m, the objective function of the materials distributor is:

$$\max\;\pi_{R}^{MS}=(b-m)[a-\alpha (w+b)-\beta e_{0}]$$ (10)

Taking the first derivative of Eq (10) with respect to b, we obtain:

$$\frac{\partial \;\pi_{R}^{MS}}{\partial b}=a-\alpha w-2\alpha b+\alpha m-\beta e_{0}$$ (11)

Further, taking the second partial derivative of Eq (11), we get $\partial^{2}\pi_{R}^{MS}/\partial b^{2}=-2\alpha <0$. Illustrating that the objective Eq (10) has a maximum value. Therefore, setting the first partial derivative of Eq (11) equal to zero, we obtain:

$$\dot{b}=\frac{a-\alpha (w-m)}{2\alpha }-\frac{\beta e_{0}}{2\alpha }$$ (12)

The objective function of the integrated service platform is:

$${max\;\pi }_{p}^{MS}=2m[a-\alpha (w+b)-\beta e_{0}]$$ (13)

Substituting $\dot{b}$ into Eq (13) and applying the first and second-order conditions for profit maximization, the solution can be obtained:

$$\dot{m}=\frac{a}{2\alpha }-\frac{w}{2}-\frac{\beta e_{0}}{2\alpha }$$ (14)

$$\ddot{b}=\frac{3a}{4\alpha }-\frac{3w}{4}-\frac{3\beta e_{0}}{4\alpha }$$ (15)

Similarly, substituting $\dot{m}\;\mathrm{and}\;\ddot{b}$ back into the profit function for the materials supplier:

$${max\;\pi }_{M}^{MS}=(w-m-c)[a-\alpha (w+b)-\beta e_{0}]$$ (16)

By substituting the maximization conditions, the equilibrium decisions for each main entity in the final platform supply chain, when they do not collaborate on carbon reduction, can be derived. When sports events materials distributor and integrated service platform serve as the respective leaders in the platform supply chain, the model construction and solution process are similar to the aforementioned procedure. The optimal decision results of the solution are presented in the Table 2.

**Table 2:** The optimal decision in the absence of low-carbon collaboration.

Decision variables	MS	RS	PS
w^0^	$\frac{2a}{3\alpha }-\frac{2\beta e_{0}}{3\alpha }+\frac{c}{3}$	$\frac{a}{4\alpha }-\frac{\beta e_{0}}{4\alpha }+\frac{3c}{4}$	$\frac{a}{2\alpha }-\frac{\beta e_{0}}{2\alpha }+\frac{c}{2}$
b^0^	$\frac{a}{4\alpha }-\frac{\beta e_{0}}{4\alpha }-\frac{c}{4}$	$\frac{2a}{3\alpha }-\frac{2\beta e_{0}}{3\alpha }-\frac{2c}{3}$	$\frac{3a}{8\alpha }-\frac{3\beta e_{0}}{8\alpha }-\frac{3c}{8}$
m^0^	$\frac{a}{6\alpha }-\frac{\beta e_{0}}{6\alpha }-\frac{c}{6}$	$\frac{a}{6\alpha }-\frac{\beta e_{0}}{6\alpha }-\frac{c}{6}$	$\frac{a}{4\alpha }-\frac{\beta e_{0}}{4\alpha }-\frac{c}{4}$
p^0^	$\frac{11a}{12\alpha }-\frac{11\beta e_{0}}{12\alpha }+\frac{c}{12}$	$\frac{11a}{12\alpha }-\frac{11\beta e_{0}}{12\alpha }+\frac{c}{12}$	$\frac{7a}{8\alpha }-\frac{7\beta e_{0}}{8\alpha }+\frac{c}{8}$
π^0^_M_	$\frac{{\left(a-c\alpha -\beta e_{0}\right)}^{2}}{24\alpha }$	$\frac{{\left(a-c\alpha -\beta e_{0}\right)}^{2}}{144\alpha }$	$\frac{{\left(a-c\alpha -\beta e_{0}\right)}^{2}}{32\alpha }$
π^0^_R_	$\frac{{\left(a-c\alpha -\beta e_{0}\right)}^{2}}{144\alpha }$	$\frac{{\left(a-c\alpha -\beta e_{0}\right)}^{2}}{24\alpha }$	$\frac{{\left(a-c\alpha -\beta e_{0}\right)}^{2}}{64\alpha }$
π^0^_P_	$\frac{{\left(a-c\alpha -\beta e_{0}\right)}^{2}}{36\alpha }$	$\frac{{\left(a-c\alpha -\beta e_{0}\right)}^{2}}{36\alpha }$	$\frac{{\left(a-c\alpha -\beta e_{0}\right)}^{2}}{16\alpha }$
π^0^_SC_	$\frac{{11(a-c\alpha -\beta e_{0})}^{2}}{144\alpha }$	$\frac{{11(a-c\alpha -\beta e_{0})}^{2}}{144\alpha }$	$\frac{{7(a-c\alpha -\beta e_{0})}^{2}}{64\alpha }$

When various entities within the sports events materials platform supply chain are subject to centralized decision-making, the objective is to maximize the overall profit of the platform supply chain. In other words, the objective function is:

$$\max\;\pi_{SC}=(p-c)(a-\alpha p-\beta e_{0})$$ (17)

#### 4.1.2. Centralized decision-making

Under centralized decision-making without low-carbon collaboration, the optimal selling price ***p*** for the materials distributor is:

$$p^{0}=\frac{a}{2\alpha }-\frac{\beta e_{0}}{2\alpha }+\frac{c}{2}$$ (18)

The overall profit of the platform supply chain is:

$${\pi^{0}}_{SC}=\frac{{\left(a-c\alpha -\beta e_{0}\right)}^{2}}{4\alpha }$$ (19)

Corollary 1:

1. ${\pi^{0}}_{M}^{MS}>{\pi^{0}}_{P}^{MS}>{\pi^{0}}_{R}^{MS};{\pi^{0}}_{R}^{RS}>{\pi^{0}}_{P}^{MS}>{\pi^{0}}_{M}^{MS};{\pi^{0}}_{P}^{PS}>{\pi^{0}}_{M}^{PS}>{\pi^{0}}_{R}^{PS}{{;\pi }^{0}}_{SC}^{MS}={\pi^{0}}_{SC}^{RS}<{\pi^{0}}_{SC}^{PS}$;
2. ${\pi^{0}}_{SC}^{MS}={\pi^{0}}_{SC}^{RS}<{\pi^{0}}_{SC}^{PS}<{\pi^{0}}_{SC}$;

Corollary 1 can be directly inferred from the contents of Table 2. Corollary 1 indicates that leaders in the platform supply chain can achieve the highest profit. Specifically, when the materials supplier dominates, its profit constitutes 54.5% of the overall profit, similar to the profit distribution ratio when the distributor is in control. Meanwhile, when the integrated service platform takes the lead, the platform’s own profit constitutes 57.1% of the overall profit. This clearly demonstrates the advantage of leaders in profit distribution, with the integrated service platform having the strongest distribution advantage. Under the dominance of the materials supplier and distributor, the overall profit of the platform supply chain is equal and lower than that under the dominance of the integrated service platform. This reflects the effective enhancement of overall profit by the unique resources and brand services of the integrated service platform. Under decentralized decision-making, the overall profit of the platform supply chain is lower than that under centralized decision-making. This indicates that, without low-carbon cooperation, centralized decision-making is more conducive to coordination between platform supply chains, leading to an overall improvement in benefits.

The rationale is that when participants in a platform-based supply chain make unified decisions, it helps to avoid resource waste and information asymmetry that arise from independent actions, thereby enhancing overall benefits. This approach facilitates centralized data analysis and decision support, enabling swift responses to market changes or crises. Moreover, centralized decision-making reduces the complexity of decisions, lowers coordination costs, and ensures consistency and coherence in decision execution. However, in the face of diverse demands and rapidly changing market conditions, centralized decision-making may struggle to obtain sufficient on-the-ground information in a timely manner, which can impair the accuracy and flexibility of decisions. In contrast, while decentralized decision-making offers advantages in flexibility and rapid response to market demands, it may lead participants to prioritize short-term gains over overall benefits, thereby diminishing collaborative potential and increasing management complexity.

### 4.2. Model analysis under low-carbon collaboration

When various entities within the sports events materials platform supply chain reach a low-carbon collaboration agreement, in order to further meet the events organizers’ low-carbon preferences, the integrated service platform considers providing carbon reduction technology support to the materials supplier to a degree denoted as *h*. At this point, the integrated service platform needs to increase its investment cost by *h*^2^*u*_*p*_/2. The platform’s investment of *h* in technical support will result in a reduction of carbon emissions per unit product, denoted as λe_Δ_, for the materials supplier. Consequently, the unit product production cost for the materials supplier becomes: $c_{\Delta }=c_{0}+t(e_{0}-e_{\Delta })=c-te_{\Delta }$. At this juncture, the profit functions for the materials supplier, materials distributor, integrated service platform, and the overall platform supply chain are transformed as follows:

$$\pi_{M}=[w-m-c+te_{\Delta }][a-\alpha (w+b)-\beta {(e}_{0}-e_{\Delta })]$$ (20)

$$\pi_{R}=(b-m)[a-\alpha (w+b)-\beta {(e}_{0}-e_{\Delta })]$$ (21)

$$\pi_{P}=2m[a-\alpha (w+b)-\beta {(e}_{0}-e_{\Delta })]-h^{2}\frac{u_{p}}{2}$$ (22)

$$\pi_{SC}=[w+b-c+te_{\Delta })[a-\alpha (w+b)-\beta {(e}_{0}-e_{\Delta })]-h^{2}\frac{u_{p}}{2}$$ (23)

#### 4.2.1. Decentralized decision-making

When the materials supplier holds dominance throughout the entire sports events materials platform supply chain, the materials distributor and integrated service platform are in an equal position. In this scenario, the materials supplier, acting as the leader in the Stackelberg game, first determines the wholesale price *w* and the optimal carbon reduction quantity *e*_Δ_ for the product. Subsequently, the integrated service platform decides its unit commission *m* and the level of investment in carbon reduction technology *h*, while the materials distributor determines the unit product market markup profit *b* (or price *p*). The obtained optimal decisions are:

$$w_{MS}^{1}=\frac{B+\alpha c}{\alpha }-\frac{AB}{2\alpha (A-t\alpha \beta +\alpha \lambda^{2}u_{p})}$$ (24)

$$m_{MS}^{1}=\frac{\lambda^{2}Bu_{p}}{2(A-t\alpha \beta +\alpha \lambda^{2}u_{p})}$$ (25)

$$h_{MS}^{1}=\frac{\beta \lambda B}{2(A-t\alpha \beta +\alpha \lambda^{2}u_{p})}$$ (26)

$${e_{△}}_{MS}^{1}=\frac{\beta B}{2(A-t\alpha \beta +\alpha \lambda^{2}u_{p})}$$ (27)

$$b_{MS}^{1}=\frac{3\alpha \lambda^{2}u_{p}B}{4\alpha (A-t\alpha \beta +\alpha \lambda^{2}u_{p})}$$ (28)

$$p_{MS}^{1}=b_{MS}^{1}+w_{MS}^{1}=\frac{a-\beta e_{0}}{\alpha }-\frac{2AB-3\alpha \lambda^{2}u_{p}B}{4\alpha (A-t\alpha \beta +\alpha \lambda^{2}u_{p})}$$ (29)

$Where\;A=2\alpha \lambda^{2}u_{p}-\beta^{2}>0;B=a-c\alpha -\beta e_{0}>0.$

At this point, the profits for the materials supplier, materials distributor, integrated service platform, and the overall platform supply chain are as follows:

$${\pi^{1}}_{M}^{MS}=\frac{\lambda^{2}B^{2}u_{p}}{8[3\alpha \lambda^{2}u_{p}-\beta (t\alpha +\beta )]}$$ (30)

$${\pi^{1}}_{R}^{MS}=\frac{\lambda^{4}B^{2}{u_{p}}^{2}}{16{\left[3\alpha \lambda^{2}u_{p}-\beta (t\alpha +\beta )\right]}^{2}}$$ (31)

$${\pi^{1}}_{P}^{MS}=\frac{\lambda^{2}B^{2}u_{p}A}{8{\left[3\alpha \lambda^{2}u_{p}-\beta (t\alpha +\beta )\right]}^{2}}$$ (32)

$${\pi^{1}}_{SC}^{MS}=\frac{\lambda^{2}B^{2}u_{p}[-2\beta (t\alpha +2\beta )+11\alpha \lambda^{2}u_{p}]}{16{\left[3\alpha \lambda^{2}u_{p}-\beta (t\alpha +\beta )\right]}^{2}}$$ (33)

Prove as follows: We use the method of backward induction to solve the problem.

The solving process is similar to the scenario where the materials supplier dominates when there is no low-carbon cooperation. The only difference is that, with the decision variable of the degree of carbon reduction technology support added, the profit function of the integrated service platform becomes a binary function. The determination of whether a maximum value exists relies on the Hessian matrix. Since each unit of carbon reduction technology support input *h* by the integrated service platform results in a carbon reduction quantity *e*_Δ_ for the materials supplier’s unit product, it can be set as *e*_Δ_ = *h*/*λ* and simultaneously substituted into Eq (23). In the end, the equilibrium decision under the dominance of materials supplier can be determined.

Similarly, equilibrium decisions under the dominance of the materials distributor (RS) and the integrated service platform (PS) can be determined. The results of carbon reduction collaboration equilibrium under different dominance structures in the decentralized scenario are summarized in Table 3.

**Table 3:** The optimal decision in low-carbon collaboration.

Decision variables	MS	RS	PS
*w* ^1^	$\frac{B+\alpha c}{\alpha }-\frac{AB}{2\alpha (A-t\alpha \beta +\alpha \lambda^{2}u_{p})}$	$\frac{B+4c\alpha }{4\alpha }-\frac{(t\alpha -\beta )(t\alpha +\beta )B}{4\alpha (C+\alpha \lambda^{2}u_{p})}$	$\frac{c\alpha (C+6\alpha \lambda^{2}u_{p}+t^{2}\alpha^{2}-\beta^{2})+\left(a-\beta e_{0})(C+6\alpha \lambda^{2}u_{p}-t^{2}\alpha^{2}+\beta^{2}\right)}{2\alpha (C+6\alpha \lambda^{2}u_{p})}$
*b* ^1^	$\frac{3\alpha \lambda^{2}u_{p}B}{4\alpha (A-t\alpha \beta +\alpha \lambda^{2}u_{p})}$	$\frac{B(C+2\alpha \lambda^{2}u_{p})}{2\alpha (C+\alpha \lambda^{2}u_{p})}$	$\frac{12B\alpha \lambda^{2}u_{p}}{4\alpha (C+6\alpha \lambda^{2}u_{p})}$
*e*_Δ_^1^	$\frac{\beta B}{2(A-t\alpha \beta +\alpha \lambda^{2}u_{p})}$	$\frac{(t\alpha +\beta )B}{2(C+\alpha \lambda^{2}u_{p})}$	$\frac{(t\alpha +\beta )B}{C+6\alpha \lambda^{2}u_{p}}$
*m* ^1^	$\frac{\lambda^{2}Bu_{p}}{2(A-t\alpha \beta +\alpha \lambda^{2}u_{p})}$	$\frac{B\lambda^{2}u_{p}}{2(C+\alpha \lambda^{2}u_{p})}$	$\frac{2\lambda^{2}Bu_{p}}{C+6\alpha \lambda^{2}u_{p}}$
*h* ^1^	$\frac{\beta \lambda B}{2(A-t\alpha \beta +\alpha \lambda^{2}u_{p})}$	$\frac{(t\alpha +\beta )B\lambda }{2(C+\alpha \lambda^{2}u_{p})}$	$\frac{(t\alpha +\beta )B\lambda }{2(C+\alpha \lambda^{2}u_{p})}$
*p* ^1^	$\frac{a-\beta e_{0}}{\alpha }-\frac{2AB-3\alpha \lambda^{2}u_{p}B}{4\alpha (A-t\alpha \beta +\alpha \lambda^{2}u_{p})}$	$\frac{B+4c\alpha }{4\alpha }+\frac{(t\alpha -\beta )(t\alpha +\beta )B+2B(C+2\alpha \lambda^{2}u_{p})}{4\alpha (C+\alpha \lambda^{2}u_{p})}$	$\frac{B12\alpha \lambda^{2}u_{p}+2c\alpha (C+6\alpha \lambda^{2}u_{p}+t^{2}\alpha^{2}-\beta^{2})+2(a-\beta e_{0})(C+6\alpha \lambda^{2}u_{p}-t^{2}\alpha^{2}+\beta^{2})}{4\alpha (C+6\alpha \lambda^{2}u_{p})}$
*π* ^1^_*M*_	$\frac{\lambda^{2}B^{2}u_{p}}{8[3\alpha \lambda^{2}u_{p}-\beta (t\alpha +\beta )]}$	$\frac{\alpha \lambda^{4}B^{2}u_{p}^{2}}{16{\left(C+\alpha \lambda^{2}u_{p}\right)}^{2}}$	$\frac{\lambda^{2}B^{2}u_{p}[-\alpha ((t-\beta )(t\alpha +\beta ))+2\alpha \lambda^{2}u_{p}]}{{\left(C+6\alpha \lambda^{2}u_{p}\right)}^{2}}$
*π* ^1^_*R*_	$\frac{\lambda^{4}B^{2}{u_{p}}^{2}}{16{\left[3\alpha \lambda^{2}u_{p}-\beta (t\alpha +\beta )\right]}^{2}}$	$\frac{\lambda^{2}B^{2}u_{p}}{8(C+\alpha \lambda^{2}u_{p})}$	$\frac{\alpha \lambda^{4}B^{2}u_{p}^{2}}{{\left(C+6\alpha \lambda^{2}u_{p}\right)}^{2}}$
*π* ^1^_*P*_	$\frac{\lambda^{2}B^{2}u_{p}A}{8{\left[3\alpha \lambda^{2}u_{p}-\beta (t\alpha +\beta )\right]}^{2}}$	$\frac{\lambda^{2}B^{2}u_{p}C}{8{\left(C+\alpha \lambda^{2}u_{p}\right)}^{2}}$	$\frac{\lambda^{2}B^{2}u_{p}}{2(C+6\alpha \lambda^{2}u_{p})}$
*π* ^1^_*SC*_	$\frac{\lambda^{2}B^{2}u_{p}[-2\beta (t\alpha +2\beta )+11\alpha \lambda^{2}u_{p}]}{16{\left[3\alpha \lambda^{2}u_{p}-\beta (t\alpha +\beta )\right]}^{2}}$	$\frac{\lambda^{2}B^{2}u_{p}[-4{\left(t\alpha +\beta \right)}^{2}+11\alpha \lambda^{2}u_{p}]}{16{\left(C+\alpha \lambda^{2}u_{p}\right)}^{2}}$	$\frac{\lambda^{2}B^{2}u_{p}[-((t\alpha +\beta )(3t\alpha +\beta -2\alpha \beta ))+14\alpha \lambda^{2}u_{p}]}{2{\left(C+6\alpha \lambda^{2}u_{p}\right)}^{2}}$

* Where A = $2\alpha \lambda^{2}u_{p}-\beta^{2}>0;\;B=a-c\alpha -\beta e_{0}>0;\;{C=2\alpha \lambda^{2}u_{p}-(t\alpha +\beta )}^{2}>0$

#### 4.2.2. Centralized decision-making

Under centralized decision-making, each platform supply chain entity aims to maximize the overall benefit of the platform supply chain. When the condition $2{\lambda^{2}u}_{p}\alpha -{\left(t\alpha +\beta \right)}^{2}=C>0$ is met, the optimal decisions and market demand for each entity in the sport events materials platform supply chain are determined as follows:

$$P^{1}=\frac{-(t\alpha +\beta )[at+(c-e_{0}t)\beta ]+{\lambda^{2}u}_{p}(a+c\alpha -e_{0}\beta )}{C}$$ (34)

$$h^{1}=\frac{(t\alpha +\beta )B\lambda }{C}$$ (35)

$$D^{1}=\frac{{\lambda^{2}\alpha u}_{p}B}{C}$$ (36)

$${\pi^{1}}_{SC}=\frac{\lambda^{2}B^{2}u_{p}[2\alpha^{2}\lambda^{2}u_{p}-{\left(t\alpha +\beta \right)}^{2}]}{2{\left[{\left(t\alpha +\beta \right)}^{2}-2\alpha \lambda^{2}u_{p}\right]}^{2}}$$ (37)

### 4.3. Comparative analysis

In this subsection, a comparative analysis of the equilibrium solutions under three distinct power structures in the two scenarios will be conducted. The aim is to uncover additional underlying properties and discuss the conditions that must be met to achieve an optimal level.

Corollary 2:

1. ${\pi^{0}}_{SC}<{\pi^{1}}_{SC},{\pi^{0}}_{M}^{MS}<{\pi^{1}}_{M}^{MS},{\pi^{0}}_{R}^{MS}<{\pi^{1}}_{R}^{MS}$, when $\beta^{2}<\frac{24\alpha \lambda^{2}u_{p}}{13},{\pi^{0}}_{P}^{MS}<{\pi^{1}}_{P}^{MS}$;
2. ${\pi^{0}}_{M}^{RS}<{\pi^{1}}_{M}^{RS},{\pi^{0}}_{R}^{RS}<{\pi^{1}}_{R}^{RS}$, when ${\beta^{2}<(t\alpha +\beta )}^{2}<\frac{3\alpha \lambda^{2}u_{p}}{2},{\pi^{0}}_{P}^{RS}<{\pi^{1}}_{P}^{RS}$;
3. ${\pi^{0}}_{R}^{PS}<{\pi^{1}}_{R}^{PS},{\pi^{0}}_{P}^{RS}<{\pi^{1}}_{P}^{RS}$, when ${\beta^{2}<(t\alpha +\beta )}^{2}<\frac{8\alpha \lambda^{2}u_{p}(1+\alpha )}{3},{\pi^{0}}_{M}^{PS}<{\pi^{1}}_{M}^{PS}$.

(for the proof, see the S1 Appendix).

Corollary 2 suggests that carbon reduction collaboration among the entities in the sports events materials platform supply chain, whether under centralized or decentralized decision-making, can to some extent promote the overall profit of the supply chain and the individual profits of each entity. However, in the platform supply chain structures where the materials supplier or distributor dominate, the profit of the integrated service platform may not always be higher when engaging in low-carbon collaboration compared to no collaboration. This is because, during low-carbon collaboration, the integrated service platform incurs costs associated with carbon reduction technology investment, impacting its profit. In contrast, under the dominance of the integrated service platform, it can leverage profit distribution advantages, resulting in higher profits under low-carbon collaboration compared to no collaboration. However, in this scenario, the profits of materials supplier may be squeezed as a consequence.

**Corollary 3:** ${\pi^{1}}_{P}^{MS}<{\pi^{1}}_{M}^{MS},{\pi^{1}}_{R}^{MS}{{<\pi }^{1}}_{M}^{MS};{\pi^{1}}_{P}^{RS}<{\pi^{1}}_{R}^{RS},{\pi^{1}}_{M}^{RS}{{<\pi }^{1}}_{R}^{RS};{\pi^{1}}_{R}^{PS}<{\pi^{1}}_{P}^{PS}$, when $\beta^{2}<24\alpha \lambda^{2}u_{p}/13,{\pi^{1}}_{M}^{PS}{{<\pi }^{1}}_{P}^{PS}$. (for the proof, see the S2 Appendix).

Corollary 3 indicates that in platform supply chain structures where different entities take the lead, the dominant party enjoys an advantage in profit distribution, similar to the conclusion drawn under decentralized decision-making without low-carbon cooperation. However, when the integrated service platform is in the leading position, its profit distribution advantage is influenced by factors such as consumer price sensitivity and consumer preferences for low-carbon products. This suggests that the bargaining power of materials supplier and distributor is higher than that of the integrated service platform. When the materials supplier and the distributor are the dominant forces, they can transfer cost pressures by reducing costs and raising prices, thereby squeezing the profit margins of the integrated service platform. In contrast, the integrated service platform primarily earns profits by charging commissions to the materials supplier and the distributor. After bearing the costs of carbon reduction technology investments independently, if the integrated service platform seeks to transfer cost pressures, it can only do so by increasing unit commissions or offering value-added services.

**Corollary 4:** ${\pi^{1}}_{SC}^{MS}<{\pi^{1}}_{SC}^{RS}<{\pi^{1}}_{SC}^{PS}$ (for the proof, see the S3 Appendix).

Corollary 4 indicates that when the integrated service platform is in control, the platform supply chain achieves the highest overall profit, while under the dominance of materials supplier, the overall profit is the lowest. This comparison underscores the superiority of the supply chain structure led by the integrated service platform in terms of economic benefits. Through multifaceted participation and service provision, the integrated service platform better meets customer needs, enhancing overall profit levels. In contrast, the narrow focus of materials supplier dominance restricts the realization of overall synergies.

The integrated service platform, through its multi-faceted engagement and comprehensive service provision, is capable of gaining deep insights into the diverse needs of customers, allowing for precise adjustments to service strategies. Additionally, centralized management and information sharing enhance supply chain transparency and coordination, facilitating automated process optimization and data integration, which in turn improves operational efficiency and overall performance. In contrast, under a supplier-dominated structure, the business focus is relatively narrow, often limited to single product transactions or specific supply chain segments, thereby constraining the realization of overall synergistic benefits.

**Corollary 5:** ${e_{△}}_{PS}^{1}<{e_{△}}_{RS}^{1};\;{e_{△}}_{MS}^{1}<{e_{△}}_{RS}^{1}$; When $2\beta^{2}>3t\alpha \beta ,{e_{△}}_{PS}^{1}<{e_{△}}_{MS}^{1}$ (for the proof, see the S3 Appendix).

Corollary 5 highlights that in a structure led by the materials distributor, the sports events materials platform supply chain achieves the highest carbon reduction level. In structures where the integrated service platform or the materials supplier dominate, the degree of carbon reduction depends on the relative magnitudes of various variables. Given the sports events materials market’s preference for low-carbon, the materials distributor directly engage with sports events organizers, acting as consumers, allowing them to swiftly grasp consumer low-carbon demands. To maximize market demand, the distributor require significant low-carbon efforts from the integrated service platform and the materials supplier. Simultaneously, being the primary source of direct orders for materials supplier, the distributor wields substantial order influence. While ensuring products meet low-carbon criteria, the distributor can influence the materials supplier production processes by adjusting orders and procurement strategies.

On a deeper level, in the distributor-dominated supply chain structure, the distributor leverages their extensive market channels and established customer relationships to rapidly respond to fluctuations in market demand. This flexibility is particularly crucial in environments where product lifecycles are short or market demands are frequently changing. However, while this distributor-dominated model excels in environments requiring high responsiveness and adaptability, it may have limitations when it comes to addressing long-term strategic goals and achieving comprehensive supply chain integration. In contrast, the supply chain dominated by an integrated service platform places a greater emphasis on efficiency and systematic integration. Through centralized management, data sharing, and process automation, the integrated service platform supports long-term strategic initiatives and facilitates comprehensive supply chain optimization and continuous improvement.

## 5. Analysis of the coordinated model introducing cost- sharing contracts

As the integrated service platform bears the entire cost of carbon reduction technology investment, its profit margin is squeezed. Therefore, it is required that the materials supplier benefiting from carbon reduction technology bear a carbon reduction technology investment cost of *φ*_*m*_(0<*φ*_*m*_<1). Assuming the dominant entity in the platform supply chain has absolute decision-making power and can determine the magnitude of *φ*_*m*_.

Under the cost-sharing contract, the objective functions of each entity in the sports events materials platform supply chain become:

$$\pi_{M}=[w-m-c+te_{\Delta }][a-\alpha (w+b)-\beta {(e}_{0}-e_{\Delta })]-{\varphi_{m}h}^{2}\frac{u_{p}}{2}$$ (38)

$$\pi_{R}=(b-m)[a-\alpha (w+b)-\beta {(e}_{0}-e_{\Delta })]$$ (39)

$$\pi_{P}=2m[a-\alpha (w+b)-\beta {(e}_{0}-e_{\Delta })]-{(1-\varphi_{m})h}^{2}\frac{u_{p}}{2}$$ (40)

$$\pi_{SC}=[w+b-c+te_{\Delta }][a-\alpha (w+b)-\beta {(e}_{0}-e_{\Delta })]-h^{2}\frac{u_{p}}{2}$$ (41)

Under the dominance of materials supplier, the materials supplier has the absolute discourse power, so they can determine the magnitude of their own proportion *φ*_*m*_ in bearing the investment cost for carbon emission reduction technology on the integrated service platform.

The profit of the materials supplier, determined through the application of backward induction method, is as follows:

$${\pi^{2}}_{M}^{MS}=\frac{\lambda^{2}{\left(-1+\varphi_{m}\right)}^{2}B^{2}u_{p}}{8\beta t\alpha [\left(-1+\varphi_{m})+\beta (-1+2\varphi_{m}\right)]+24\alpha \lambda^{2}{\left(-1+\varphi_{m}\right)}^{2}u_{p}}$$ (42)

$Where\;2\alpha \lambda^{2}u_{p}(1-\varphi_{m})-\beta^{2}>0.$

The materials supplier, acting as the leading entity, can determine the allocation ratio *φ*_*m*_ of carbon reduction technology investment costs based on the condition of profit maximization.


$$\varphi_{m}=\frac{t\alpha }{t\alpha +2\beta }$$ (43)

Substituting *φ*_*m*_ into the equilibrium decisions of each entity and the profits of various entities in the platform supply chain, the final equilibrium decisions and optimal profits are obtained as follows:

$$b_{CMS}^{2}=\frac{3B\lambda^{2}u_{p}}{12\alpha \lambda^{2}u_{p}{-(t\alpha +2\beta )}^{2}}$$ (44)

$$w_{CMS}^{2}=\frac{(t\alpha +2\beta )[c\alpha \beta +a(t\alpha +\beta )-\beta (t\alpha +\beta )e_{0}]-4\alpha \lambda^{2}(2a+c\alpha -2\beta e_{0})u_{p}}{\alpha [{\left(t\alpha +2\beta \right)}^{2}-12\alpha \lambda^{2}u_{p}]}$$ (45)

$${e_{△}}_{CMS}^{2}=\frac{B(t\alpha +2\beta )}{12\alpha \lambda^{2}u_{p}{-(t\alpha +2\beta )}^{2}}$$ (46)

$$h_{CMS}^{2}=\frac{B\lambda (t\alpha +2\beta )}{12\alpha \lambda^{2}u_{p}{-(t\alpha +2\beta )}^{2}}$$ (47)

$$m_{CMS}^{2}=\frac{{2\lambda }^{2}Bu_{p}}{12\alpha \lambda^{2}u_{p}{-(t\alpha +2\beta )}^{2}}$$ (48)

$$p_{CMS}^{2}=b_{CMS}^{2}+w_{CMS}^{2}=\frac{\begin{array}{c} \left(t\alpha +2\beta )[c\alpha \beta +a(t\alpha +\beta )-\beta (t\alpha +\beta )e_{0}\right]\\ -\alpha \lambda^{2}(11a+c\alpha -11\beta e_{0})u_{p} \end{array}}{\alpha [{\left(t\alpha +2\beta \right)}^{2}-12\alpha \lambda^{2}u_{p}]}$$ (49)

$${\pi^{2}}_{M}^{CMS}=\frac{\lambda^{2}B^{2}u_{p}}{24\alpha \lambda^{2}u_{p}{-2(t\alpha +2\beta )}^{2}}$$ (50)

$${\pi^{2}}_{R}^{CMS}=\frac{\alpha \lambda^{4}B^{2}{u_{p}}^{2}}{{\left[{\left(t\alpha +2\beta \right)}^{2}-12\alpha \lambda^{2}u_{p}\right]}^{2}}$$ (51)

$${\pi^{2}}_{P}^{CMS}=\frac{\lambda^{2}B^{2}u_{p}[-\beta (t\alpha +2\beta )+4\alpha \lambda^{2}u_{p}]}{{\left[{\left(t\alpha +2\beta \right)}^{2}-12\alpha \lambda^{2}u_{p}\right]}^{2}}$$ (52)

$${\pi^{2}}_{SC}^{CMS}=\frac{\lambda^{2}B^{2}u_{p}[22\alpha \lambda^{2}u_{p}-(t\alpha +2\beta )(t\alpha +4\beta )]}{2{\left[{\left(t\alpha +2\beta \right)}^{2}-12\alpha \lambda^{2}u_{p}\right]}^{2}}$$ (53)

Similarly, cost-sharing contract decisions under distributor dominance and integrated service platform dominance can be derived.

$When\;{\alpha \lambda^{2}u_{p}-(t\alpha +\beta )}^{2}>0,$


$$\varphi_{m}=\frac{1}{2}$$ (54)

$$b_{CRS}^{2}=\frac{B[-5{\left(t\alpha +\beta \right)}^{2}+8\alpha \lambda^{2}u_{p}]}{4\alpha [3\alpha \lambda^{2}u_{p}-2{\left(t\alpha +\beta \right)}^{2}]}$$ (55)

$$w_{CRS}^{2}=\frac{\begin{array}{c} 3\alpha (a+3c\alpha -e_{0}\beta )\lambda^{2}u_{p}-(t\alpha +\beta )[a(3t\alpha +\beta )-\\ e_{0}\beta (3t\alpha +\beta )+c\alpha (5t\alpha +7\beta )] \end{array}}{4\alpha [3\alpha \lambda^{2}u_{p}-2{\left(t\alpha +\beta \right)}^{2}]}$$ (56)

$${e_{△}}_{CRS}^{2}=\frac{B(t\alpha +\beta )}{6\alpha \lambda^{2}u_{p}-4{\left(t\alpha +\beta \right)}^{2}}$$ (57)

$$h_{CRS}^{2}=\frac{B\lambda (t\alpha +\beta )}{6\alpha \lambda^{2}u_{p}-4{\left(t\alpha +\beta \right)}^{2}}$$ (58)

$$m_{CRS}^{2}=\frac{BC}{4\alpha [3\alpha \lambda^{2}u_{p}-2{\left(t\alpha +\beta \right)}^{2}]}$$ (59)

$$p_{CRS}^{2}=b_{CRS}^{2}+w_{CRS}^{2}=\frac{\begin{array}{c} \alpha (11a+c\alpha -11e_{0}\beta )\lambda^{2}u_{p}-\\ 2(t\alpha +\beta )[a(4t\alpha +3\beta )+\beta (c\alpha -4e_{0}t\alpha -3e_{0}\beta )] \end{array}}{4\alpha [3\alpha \lambda^{2}u_{p}-2{\left(t\alpha +\beta \right)}^{2}]}$$ (60)

$${\pi^{2}}_{M}^{CRS}=\frac{\lambda^{2}B^{2}u_{p}[\alpha \lambda^{2}u_{p}-{\left(t\alpha +\beta \right)}^{2}]}{16{\left[2{\left(t\alpha +\beta \right)}^{2}-3\alpha \lambda^{2}u_{p}\right]}^{2}}$$ (61)

$${\pi^{2}}_{R}^{CRS}=\frac{\lambda^{2}B^{2}u_{p}}{8[3\alpha \lambda^{2}u_{p}-2{\left(t\alpha +\beta \right)}^{2}]}$$ (62)

$${\pi^{2}}_{P}^{CRS}=\frac{\lambda^{2}B^{2}u_{p}[-3{\left(t\alpha +\beta \right)}^{2}+4\alpha \lambda^{2}u_{p}]}{16{\left[2{\left(t\alpha +\beta \right)}^{2}-3\alpha \lambda^{2}u_{p}\right]}^{2}}$$ (63)

$${\pi^{2}}_{SC}^{CMS}=\frac{\lambda^{2}B^{2}u_{p}[22\alpha \lambda^{2}u_{p}-(t\alpha +2\beta )(t\alpha +4\beta )]}{2{\left[{\left(t\alpha +2\beta \right)}^{2}-12\alpha \lambda^{2}u_{p}\right]}^{2}}$$ (64)

$When\;{\left[8\alpha \lambda^{2}u_{p}-(t\alpha +\beta \right)}^{2}]/3>0,$


$$\varphi_{m}=\frac{2}{3}$$ (65)

$$b_{CPS}^{2}=\frac{3B[-{\left(t\alpha +\beta \right)}^{2}+8\alpha \lambda^{2}u_{p}]}{2\alpha [32\alpha \lambda^{2}u_{p}-9{\left(t\alpha +\beta \right)}^{2}]}$$ (66)

$$w_{CPS}^{2}=\frac{\begin{array}{c} 32\alpha \lambda^{2}(a+c\alpha -\beta e_{0})u_{p}-\\ 3(t\alpha +\beta )[a(5t\alpha +\beta )+c\alpha (t\alpha +5\beta )-\beta (5t\alpha +\beta )e_{0}] \end{array}}{2\alpha [32\alpha \lambda^{2}u_{p}-9{\left(t\alpha +\beta \right)}^{2}]}$$ (67)

$${e_{△}}_{CPS}^{2}=\frac{6B(t\alpha +\beta )}{32\alpha \lambda^{2}u_{p}-9{\left(t\alpha +\beta \right)}^{2}}$$ (68)

$$h_{CPS}^{2}=\frac{6\lambda B(t\alpha +\beta )}{32\alpha \lambda^{2}u_{p}-9{\left(t\alpha +\beta \right)}^{2}}$$ (69)

$$m_{CPS}^{2}=\frac{B(16\alpha \lambda^{2}u_{p}-3{\left(t\alpha +\beta \right)}^{2})}{2\alpha [32\alpha \lambda^{2}u_{p}-9{\left(t\alpha +\beta \right)}^{2}]}$$ (70)

$$p_{CPS}^{2}=b_{CPS}^{2}+w_{CPS}^{2}=\frac{\begin{array}{c} 4\alpha \lambda^{2}(7a+c\alpha -7\beta e_{0})u_{p}-\\ 3(t\alpha +\beta )[2c\alpha \beta +a(3t\alpha +\beta )-\beta (3t\alpha +\beta )e_{0}] \end{array}}{\alpha [32\alpha \lambda^{2}u_{p}-9{\left(t\alpha +\beta \right)}^{2}]}$$ (71)

$${\pi^{2}}_{M}^{CPS}=\frac{4\lambda^{2}B^{2}u_{p}[8\alpha \lambda^{2}u_{p}{-3(t\alpha +\beta )}^{2}]}{{\left[9{\left(t\alpha +\beta \right)}^{2}-32\alpha \lambda^{2}u_{p}\right]}^{2}}$$ (72)

$${\pi^{2}}_{R}^{CPS}=\frac{16{\alpha \lambda }^{4}B^{2}{u_{p}}^{2}}{{\left[9{\left(t\alpha +\beta \right)}^{2}-32\alpha \lambda^{2}u_{p}\right]}^{2}}$$ (73)

$${\pi^{2}}_{P}^{CPS}=\frac{2\lambda^{2}B^{2}u_{p}}{32\alpha \lambda^{2}u_{p}-9{\left(t\alpha +\beta \right)}^{2}}$$ (74)

$${\pi^{2}}_{SC}^{CPS}=\frac{2\lambda^{2}B^{2}u_{p}[56\alpha \lambda^{2}u_{p}-15{\left(t\alpha +\beta \right)}^{2}]}{{\left[9{\left(t\alpha +\beta \right)}^{2}-32\alpha \lambda^{2}u_{p}\right]}^{2}}$$ (75)

## 6. Numerical analysis

In this section, we use MATLAB R2022b software to perform a numerical analysis and comparative study of the equilibrium outcomes under different decision-making models. Considering the previously mentioned conditions and constraints, let $a=200,e_{0}=8,t=1,c=2,\alpha =0.8,u_{p}=10$. We explore the impact of the sports events organizers’ low-carbon preference level β, the carbon reduction impact coefficient λ of platform technology investment, and their combined effects on the profits of various entities in the sports events materials platform supply chain, as well as the impact on product carbon reduction amounts in different scenarios. Finally, the effectiveness of the cost-sharing contract is validated.

### 6.1. The influence of β on the profits of each entity and carbon reduction

**Fig 1:**
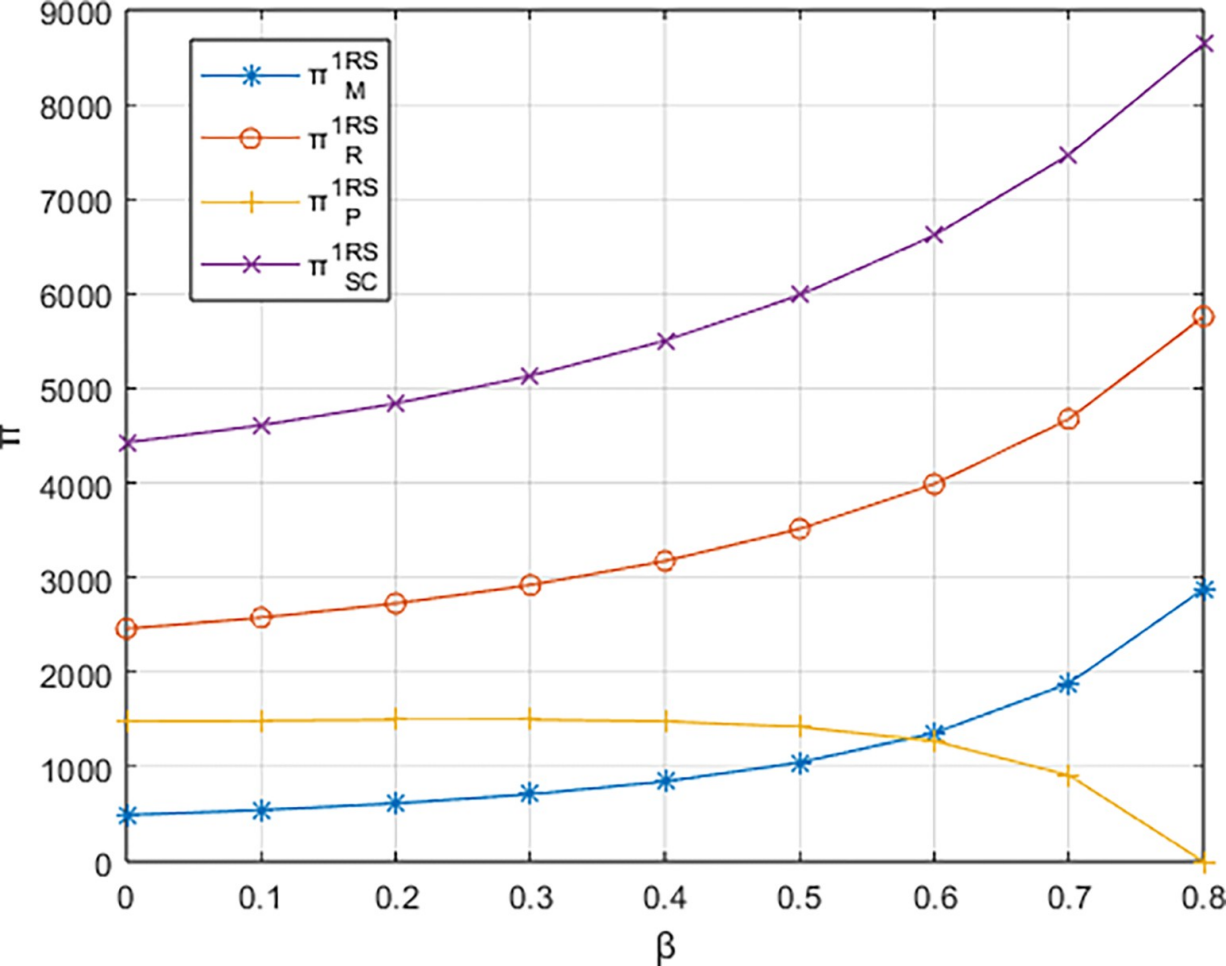
The impact of β on profits of various entities under the dominance of the materials supplier.

**Fig 2:**
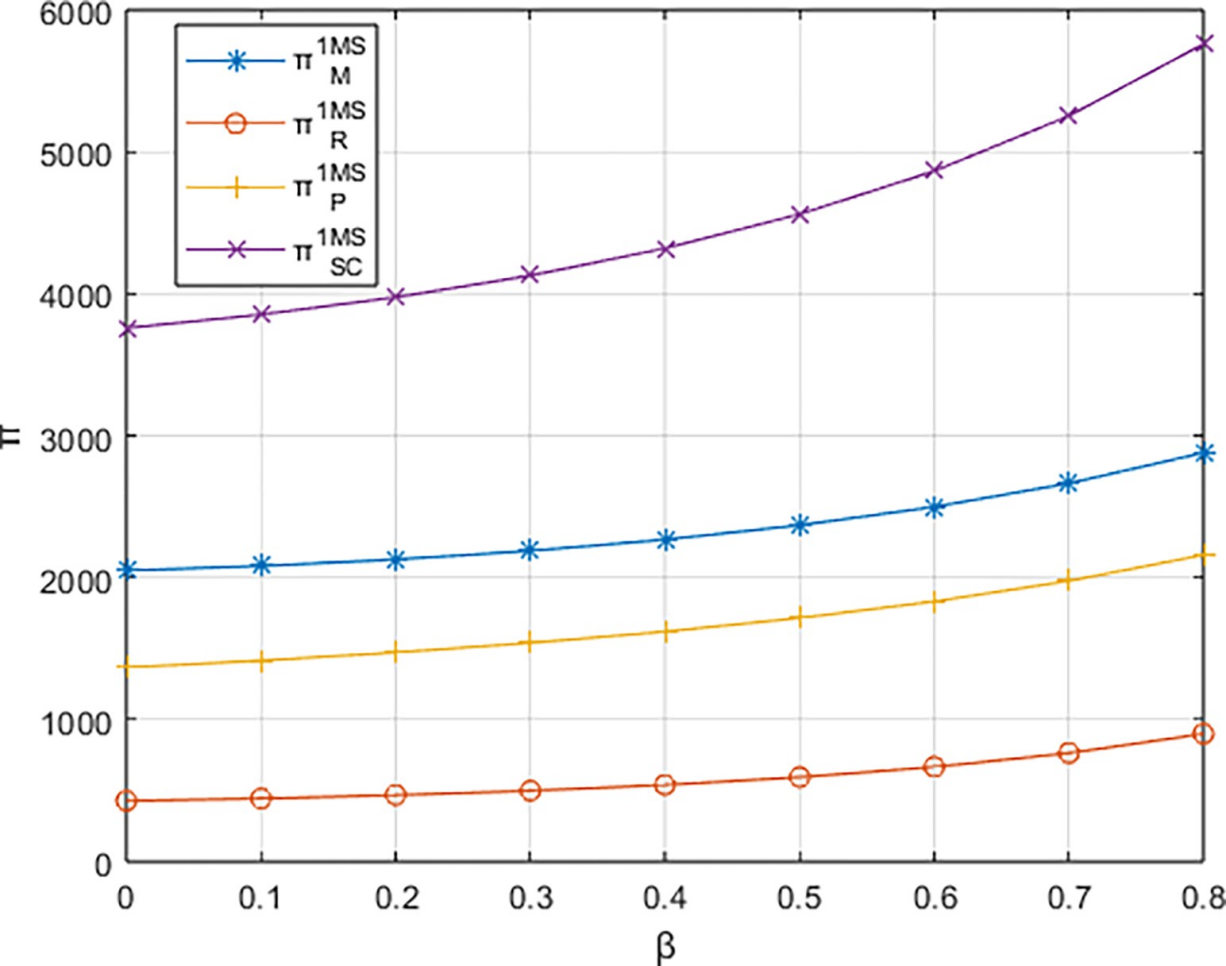
The impact of β on profits of various entities under the dominance of the materials distributor.

**Fig 3:**
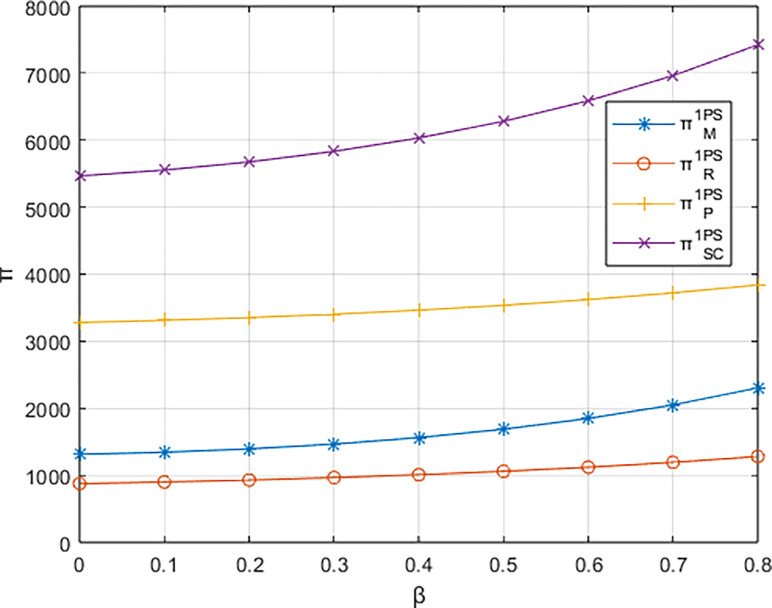
The impact of β on profits of various entities under the dominance of the integrated service platform.

Letting *λ* = 0.4 and considering *β*∈[0,0.8], the variations in profits for each entity are illustrated in Figs 1–3. From the figures, it can be observed that each entity in the platform supply chain occupies a profit distribution advantage under its respective dominant structure, confirming Corollary 3. Under the dominance of the materials supplier and the dominance of the integrated service platform, the profits of each entity in the platform supply chain, as well as the overall profit of the platform supply chain, increase with the increasing low-carbon preference level of the sports events organizers. However, under the dominance of the materials distributor, the profit of the integrated service platform decreases with the increasing low-carbon preference level, and the greater the low-carbon preference level, the faster the decline in the profit of the integrated service platform.

**Fig 4:**
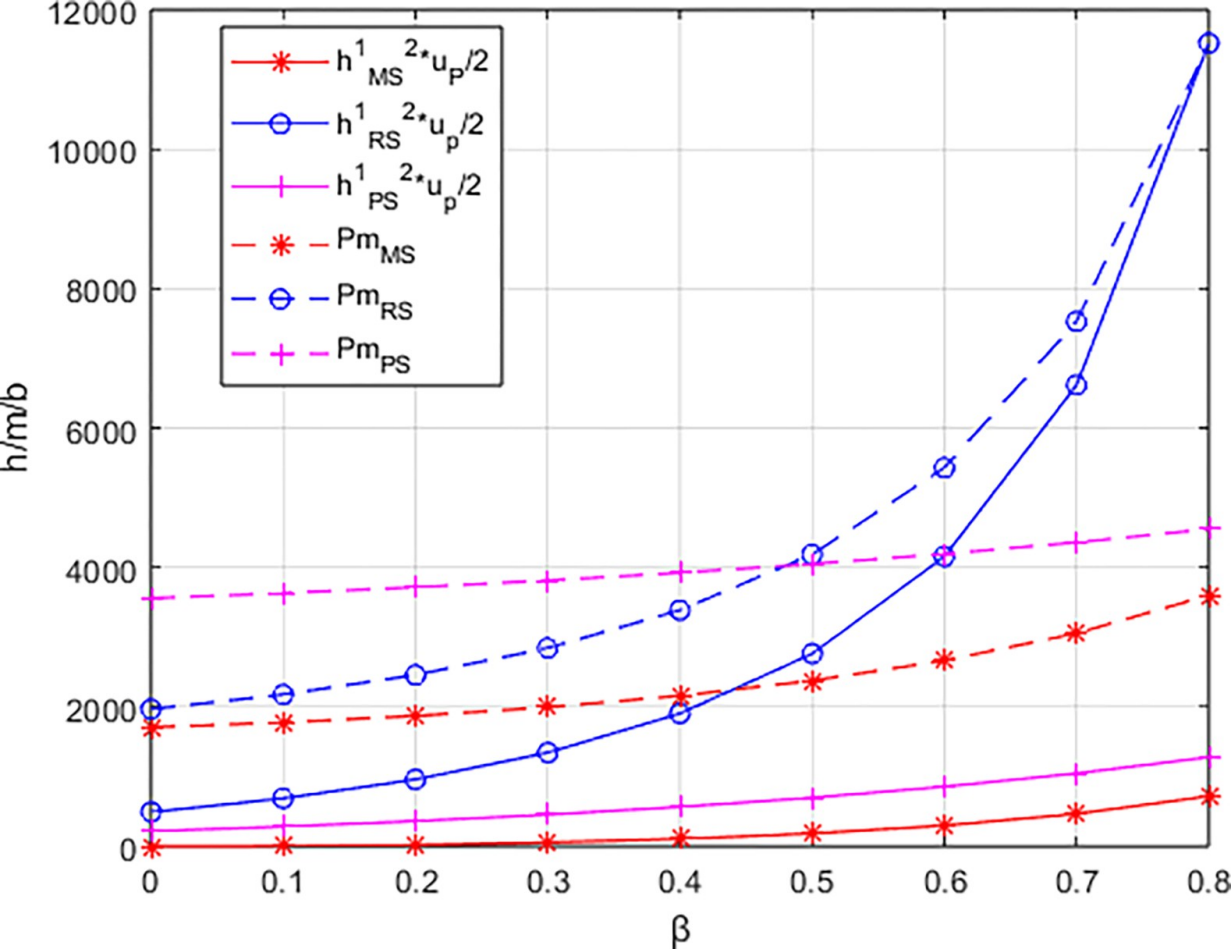
The impact of β on the investment cost of low-carbon innovation technologies and commission revenue (Pm) for the platform.

From Fig 4, it can be observed that the rate of increase in the carbon reduction technology investment cost for the integrated service platform under the dominance of the materials distributor is significantly higher than the growth rates under the dominance of the materials supplier and the dominance of the integrated service platform. When the growth rate of the platform’s carbon reduction technology investment cost exceeds the growth rate of income brought by commissions, the profit margin of the integrated service platform (highlighted by the blue-shaded area in the Fig 4 becomes progressively smaller. Consequently, the profit of the integrated service platform decreases with the increasing low-carbon preference of the sports events organizers. In contrast, under the dominance of materials suppliers and the dominance of the integrated service platform, when the increase in platform commission income is greater than the increase in technology investment costs (highlighted by the alternating purple and red-shaded areas in the Fig 4, the profit margin of the integrated service platform gradually expands. In other words, platform profit increases with the growing low-carbon preference of the sports events organizers.

**Fig 5:**
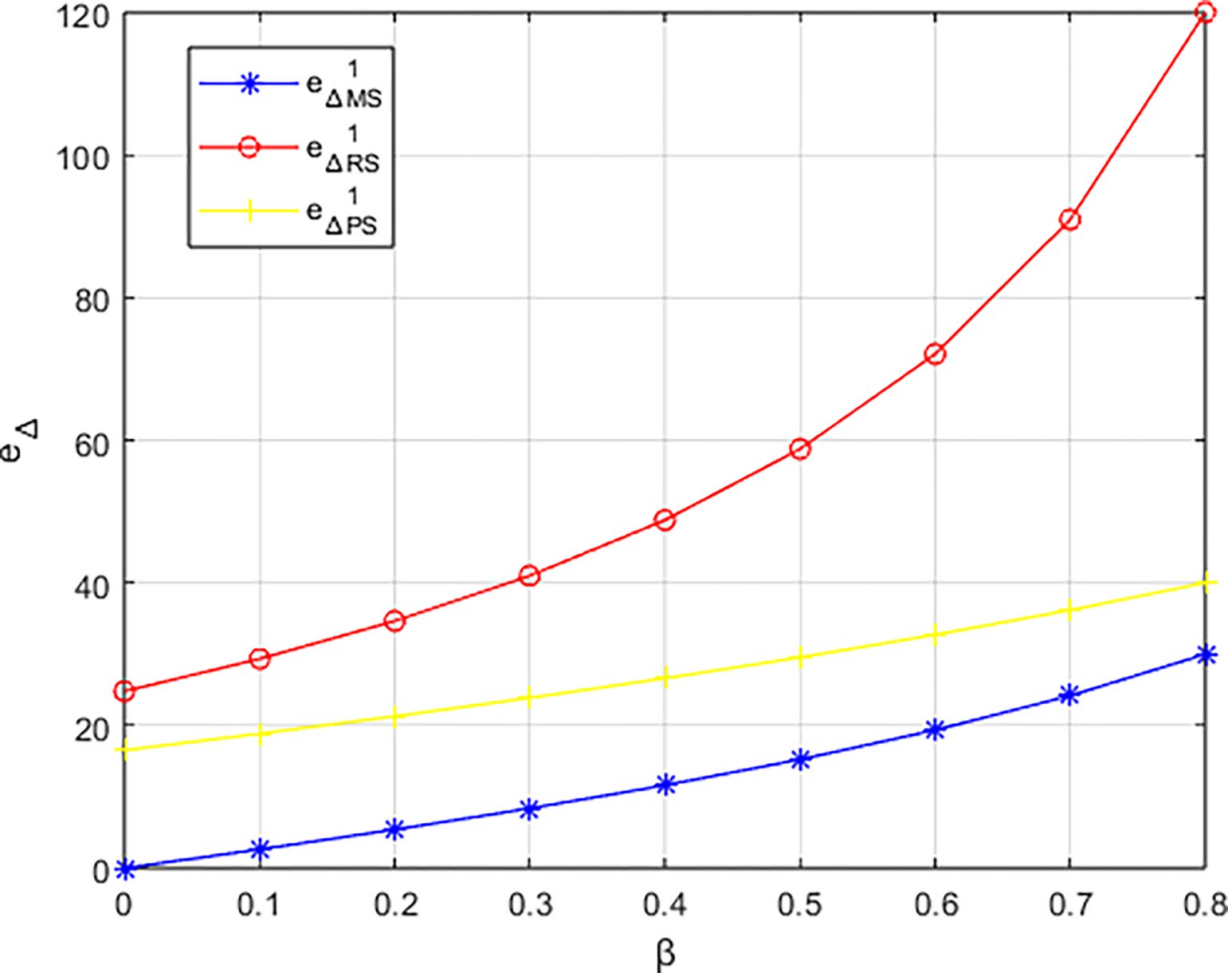
The impact of *β* on carbon emissions reduction e_Δ_.

The variation of carbon emissions reduction *e*_Δ_ under three structures with the changing preferences of the events organizing committee for low-carbon initiatives is illustrated in Fig 5. In the structure dominated by the materials distributor, the carbon reduction level exceeds that of the other two dominant structures, consistent with the conclusion in Corollary 5. Across all three structures, the carbon reduction level is positively correlated with the events organizing committee’s preference for low-carbon practices, with the greatest increase observed in the structure dominated by the materials distributor. This indicates that consumer preferences for low-carbon products can stimulate various entities within the platform’s supply chain to make efforts in carbon reduction to meet market demands. In the sports events materials platform supply chain, enterprises adapt to market demands and price signals by engaging in low-carbon collaborations to meet consumers’ demands for low-carbon products.

### 6.2. The influence of λ on the profits of each entity and carbon reduction

**Fig 6:**
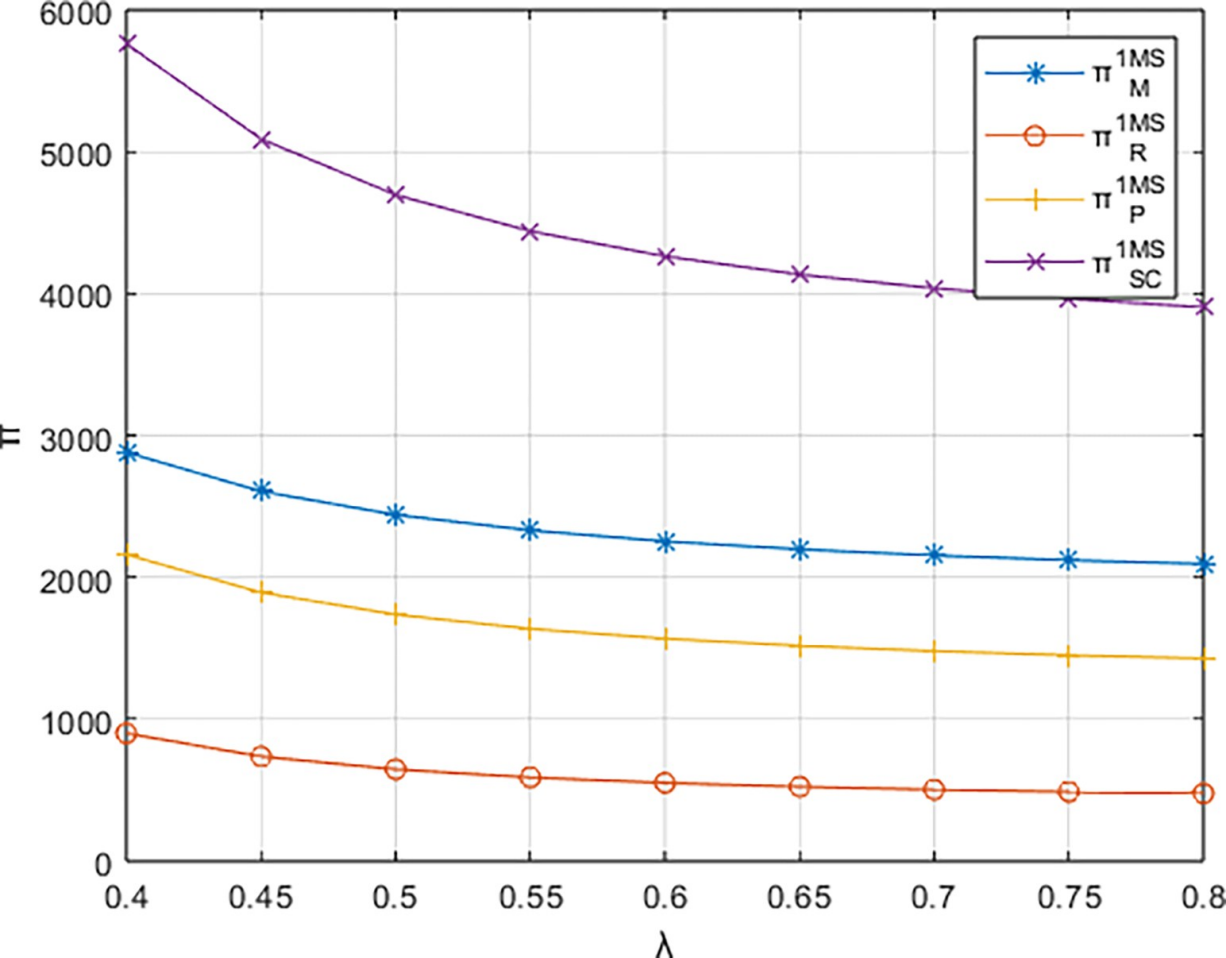
The impact of λ on profits of various entities under the dominance of the materials supplier.

**Fig 7:**
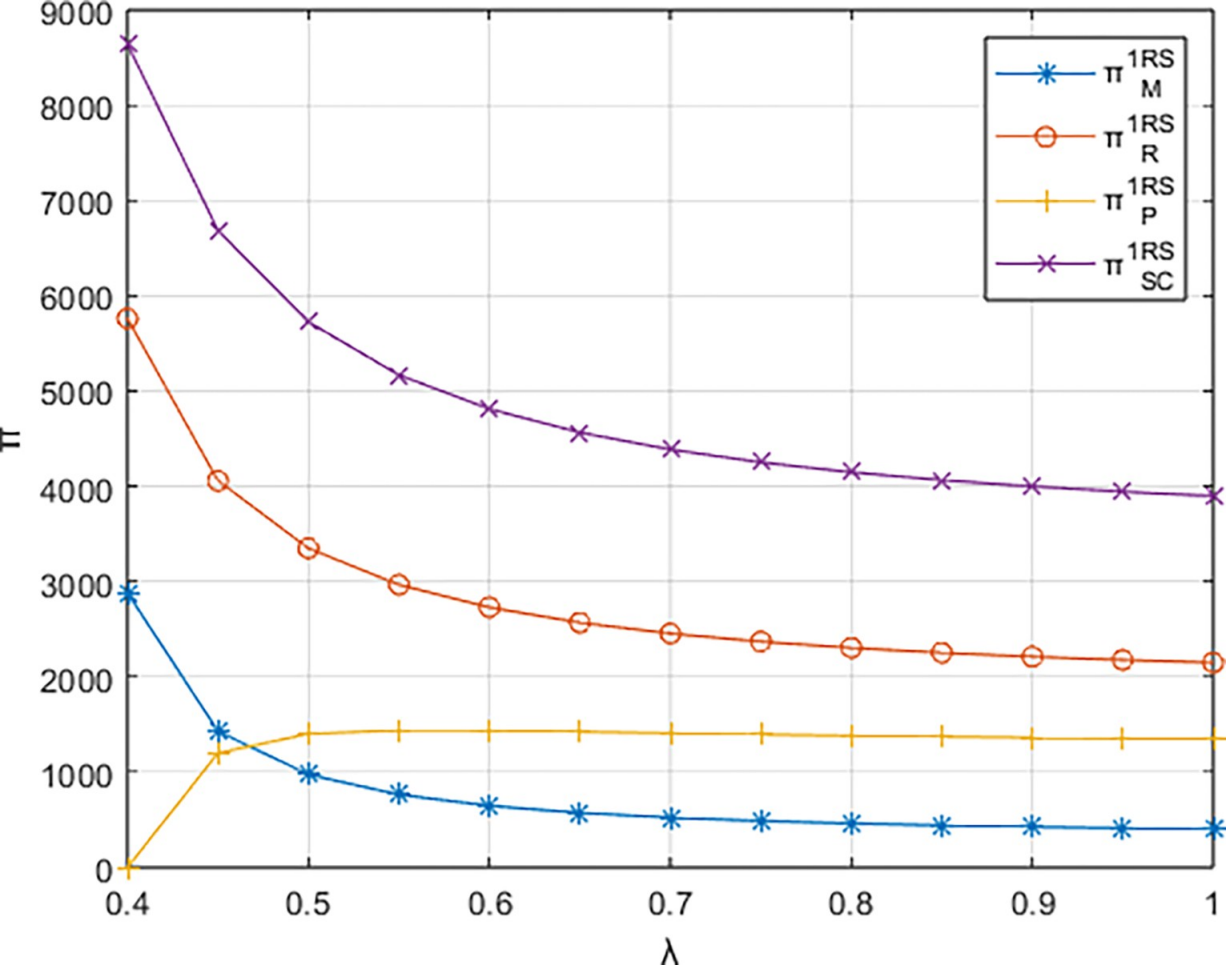
The impact of λ on profits of various entities under the dominance of the materials distributor.

**Fig 8:**
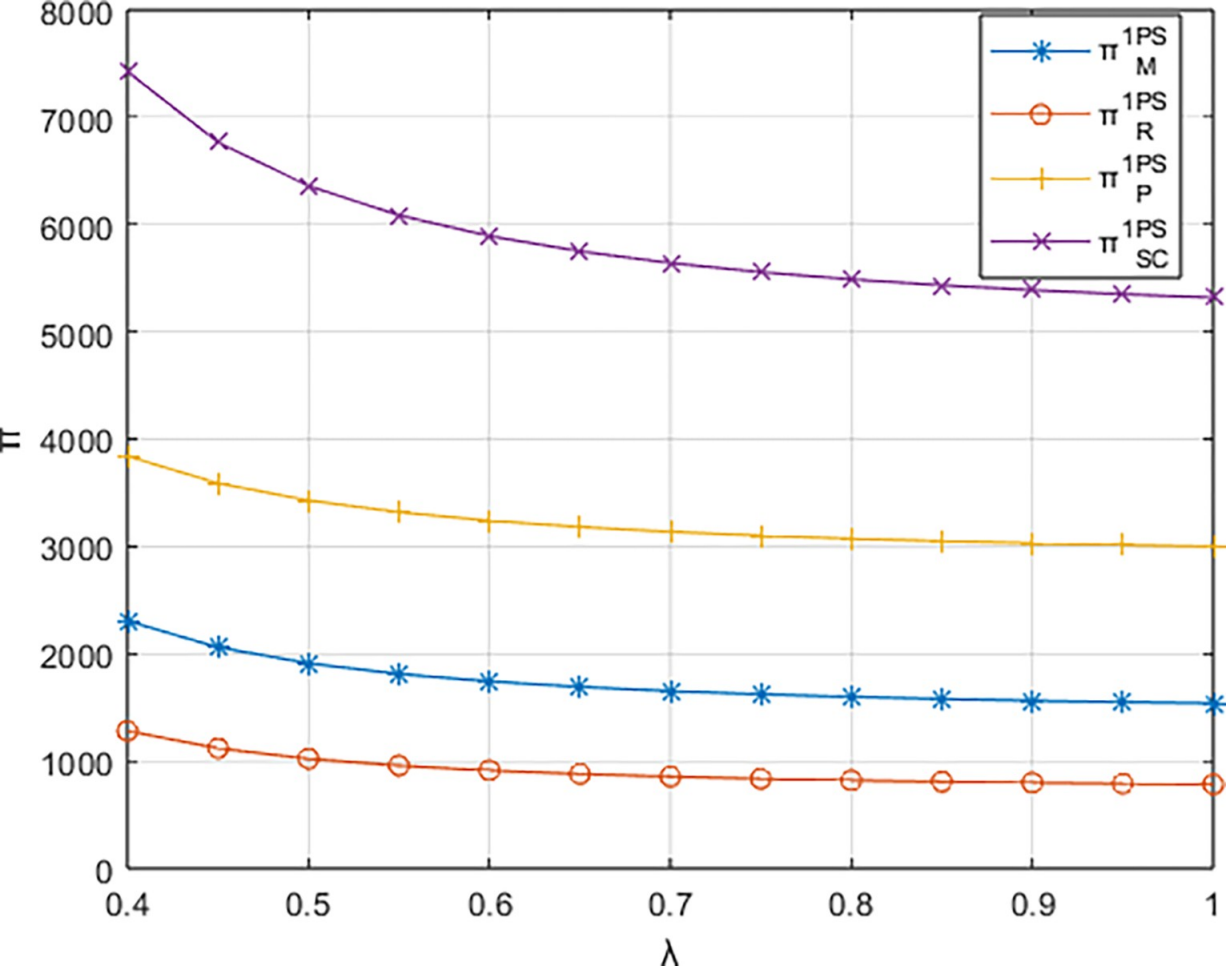
The impact of λ on profits of various entities under the dominance of the integrated service platform.

Setting *β* = 0.8 and considering *λ* ∈ [0.4,1], the variations in profits for each entity are depicted in Figs 6–8. The impact of the platform’s technological investment carbon reduction coefficient on the profits of various entities stands in direct contrast to the influence of the events organizing committee’s low-carbon preferences on entity profits.

**Fig 9:**
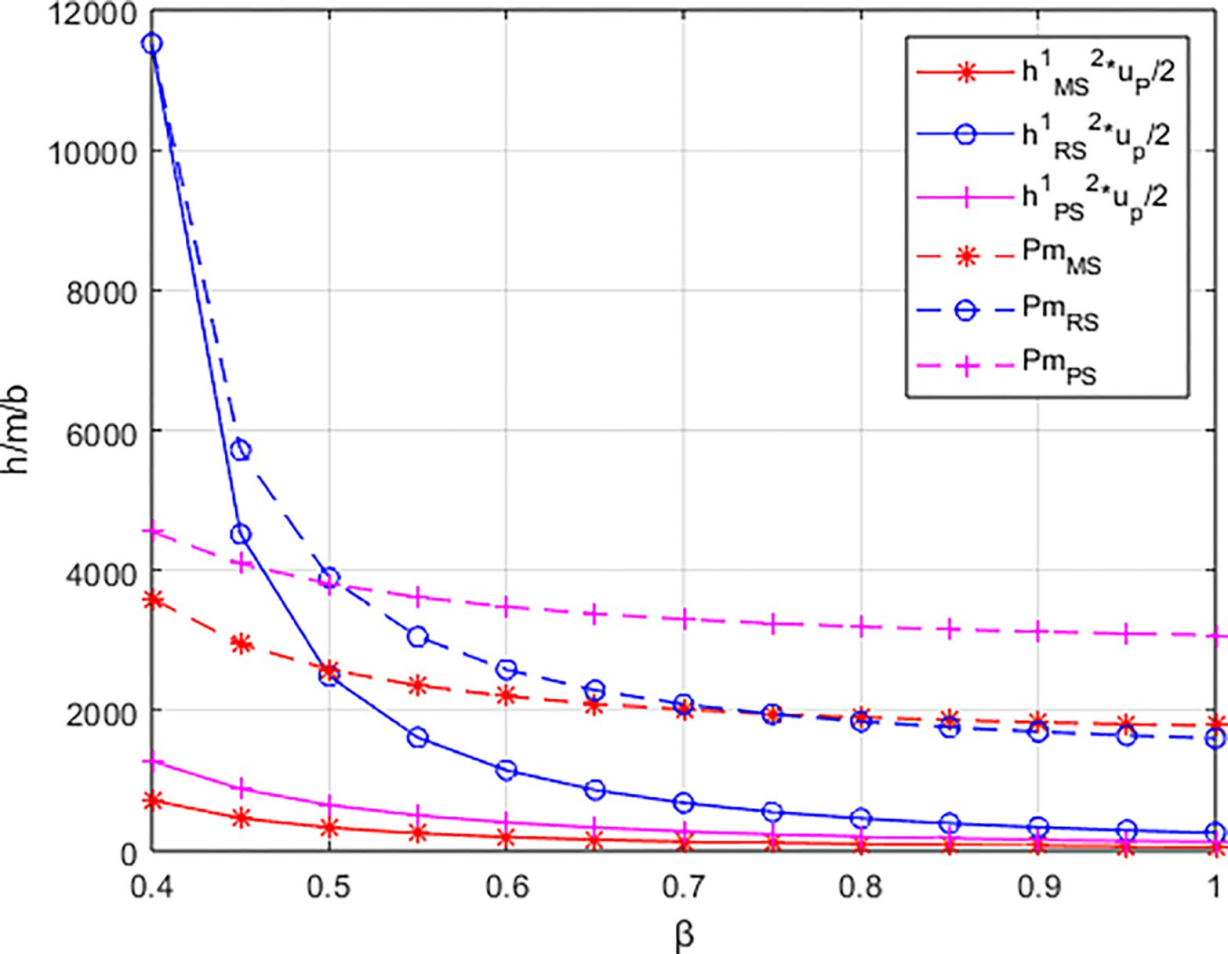
The impact of λ on the investment cost of low-carbon innovation technologies and commission revenue (Pm) for the platform.

Due to the correlation between the platform’s carbon reduction technology input coefficient and the carbon reduction of materials supplier products, a larger coefficient results in a smaller carbon reduction *e*_Δ_ under the same platform carbon reduction technology input. This leads to increased supplier costs and decreased market demand. In the structure led by the materials distributor, the decreasing rate of the integrated service platform commission income is lower than the decreasing rate of technology input costs, gradually expanding the profit margin of the integrated service platform (as indicated by the blue line segments in the Fig 9, thus proportional to the platform’s carbon reduction technology input coefficient.

**Fig 10:**
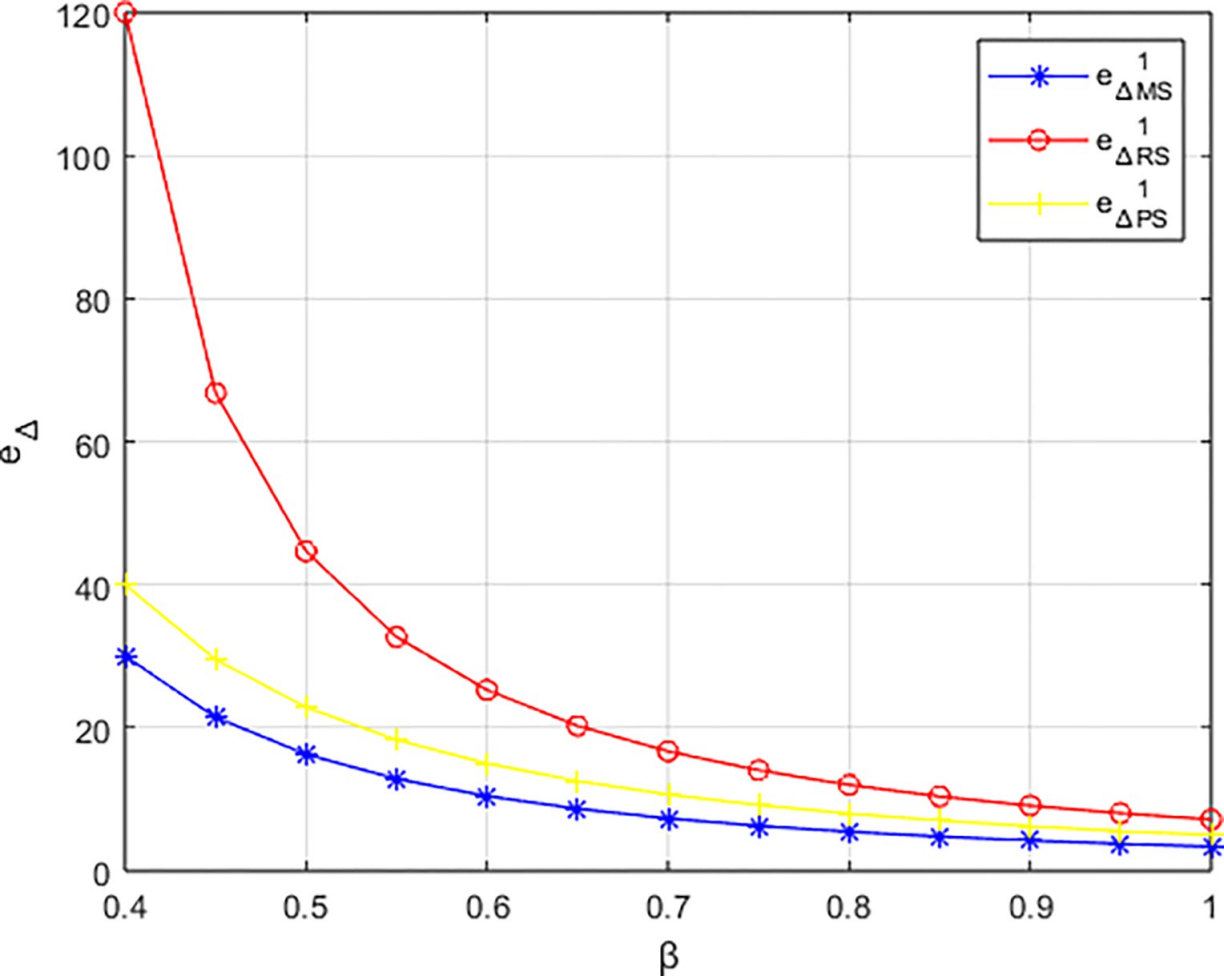
The impact of *λ* on carbon emissions reduction *e*_Δ_.

The carbon emission reduction *e*_Δ_ under three different structures varies with the changing preferences of the events organizing committee for low-carbon initiatives, as illustrated in Fig 10. Similar to the impact of low-carbon preferences on carbon emission reduction, optimal carbon reduction can be achieved under the distributor-dominated structure. However, the carbon emission reduction for products under all three structures decreases as the carbon reduction impact coefficient *λ* of platform technology investment increases, consistent with the analysis presented earlier.

### 6.3. The joint impact of β and λ on the profits of each entity

**Fig 11:**
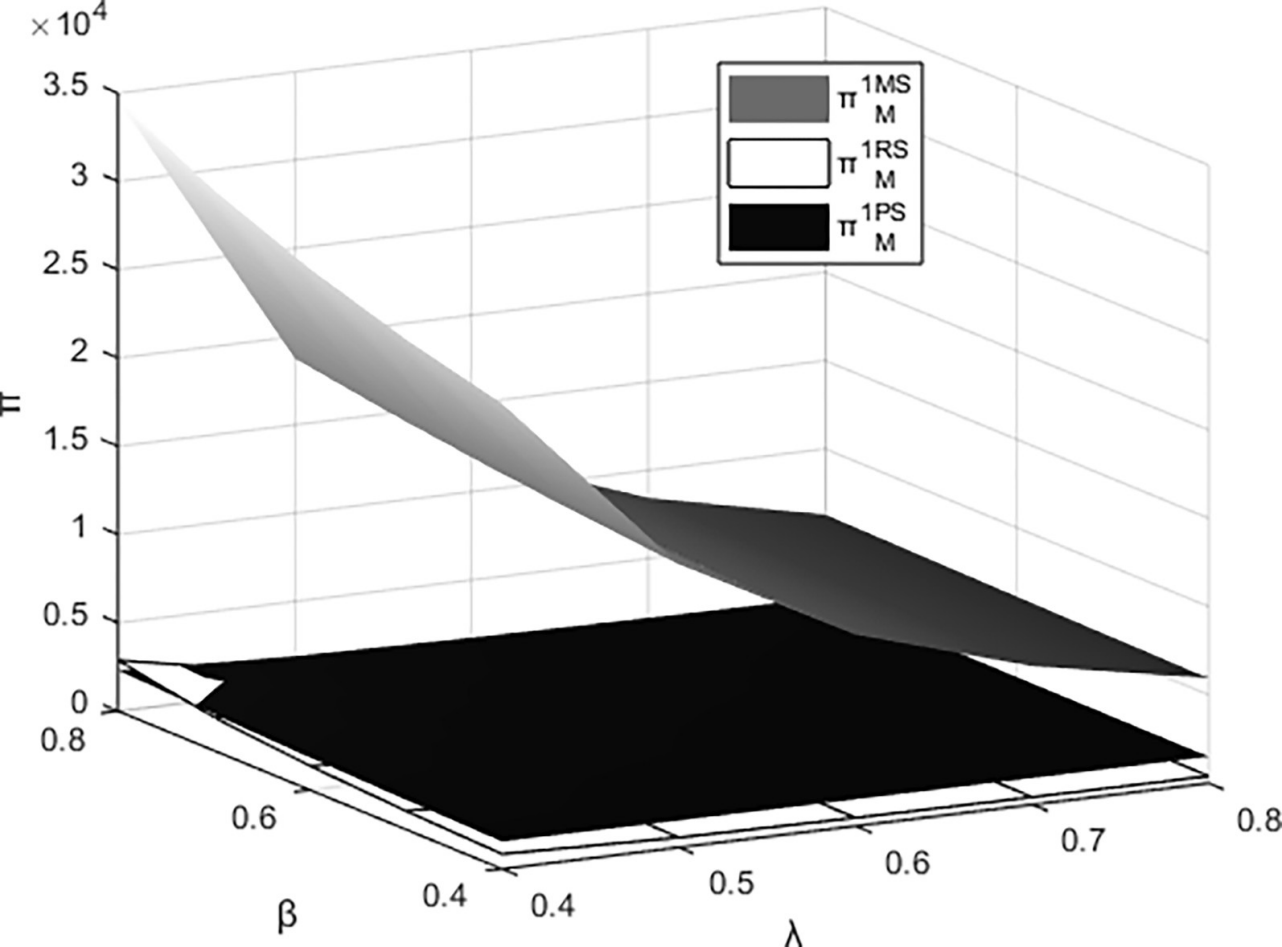
The impact of β and λ on the profitability of the materials supplier.

**Fig 12:**
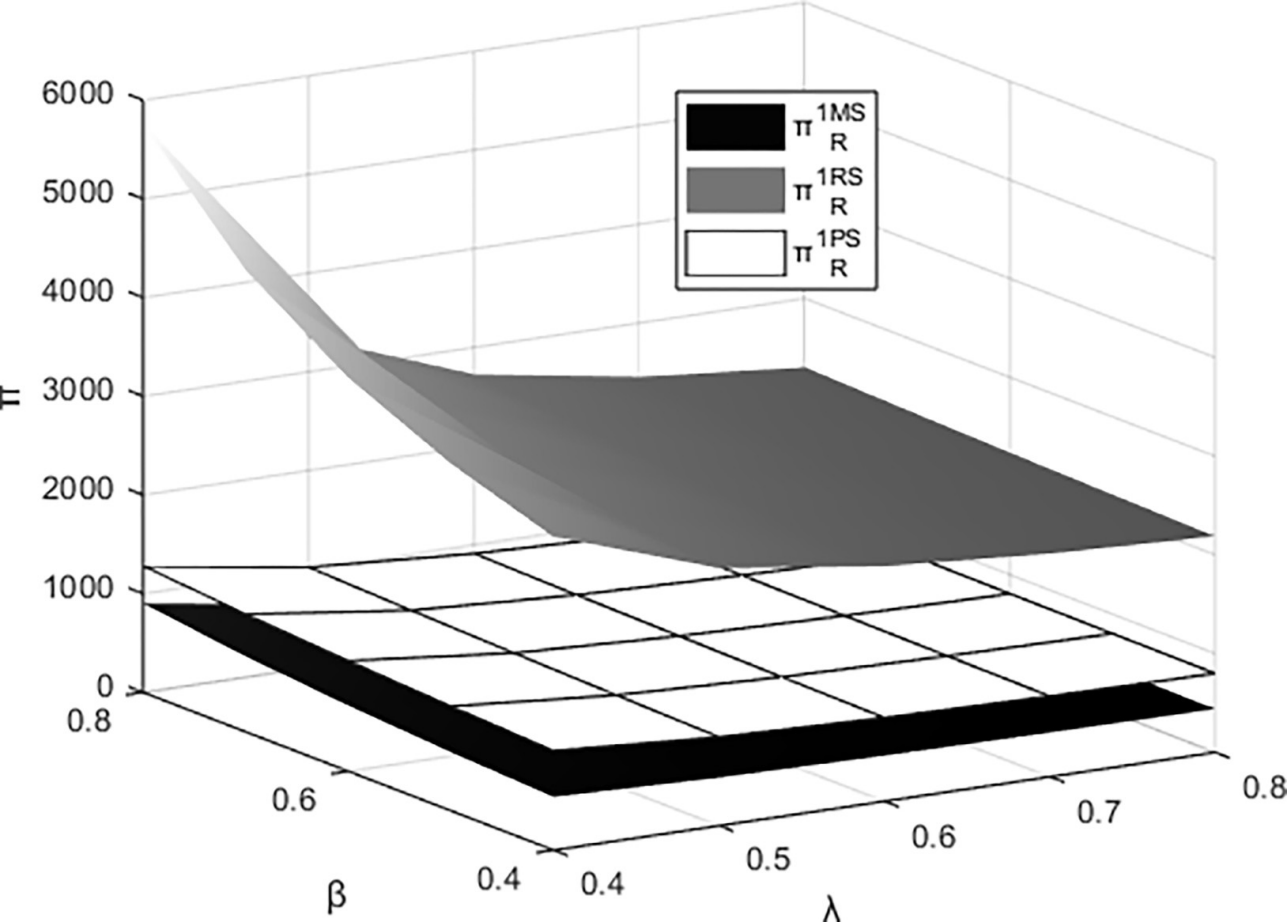
The impact of β and λ on the profitability of the materials distributor.

**Fig 13:**
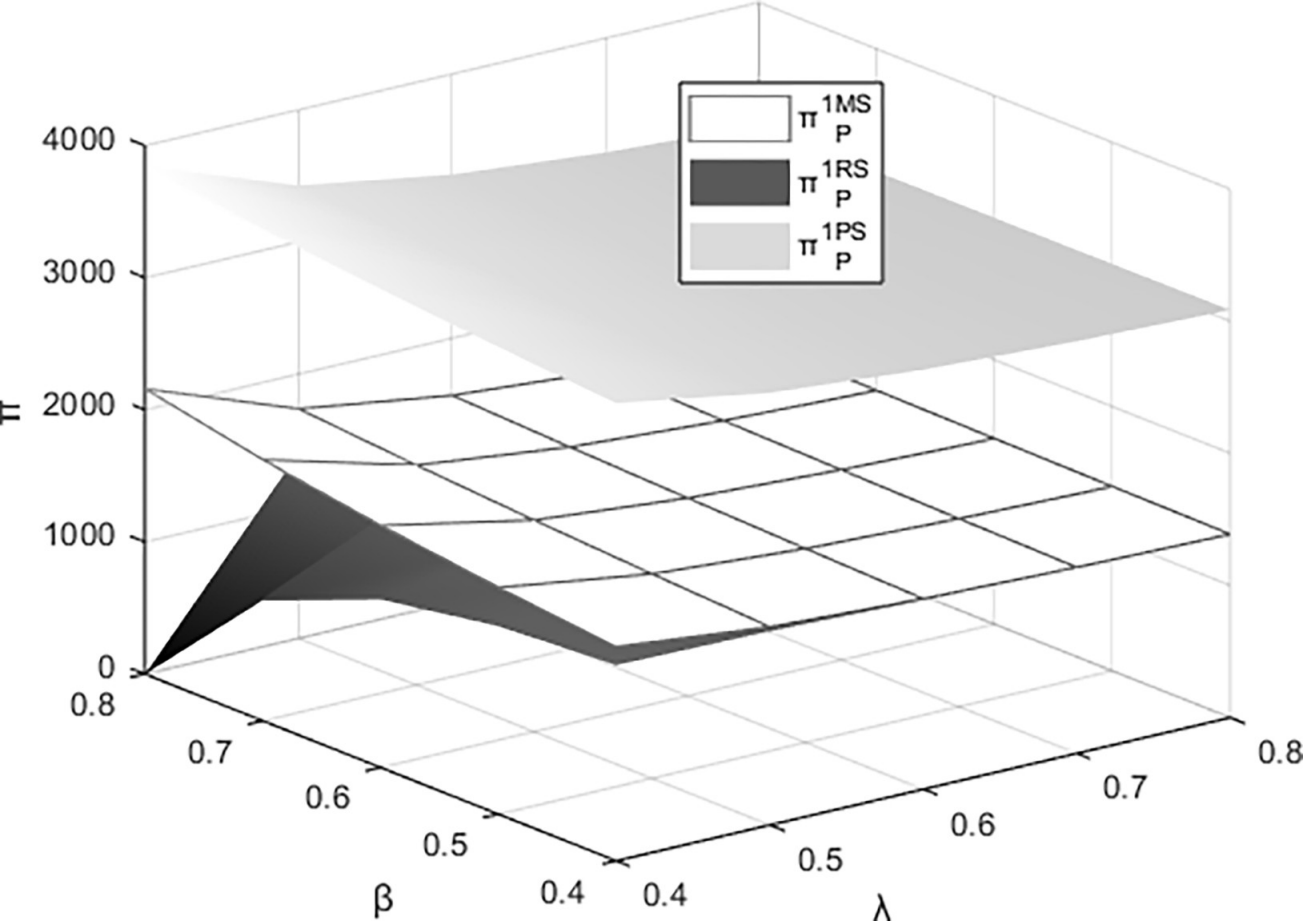
The impact of β and λ on the profits of the integrated service platform.

**Fig 14:**
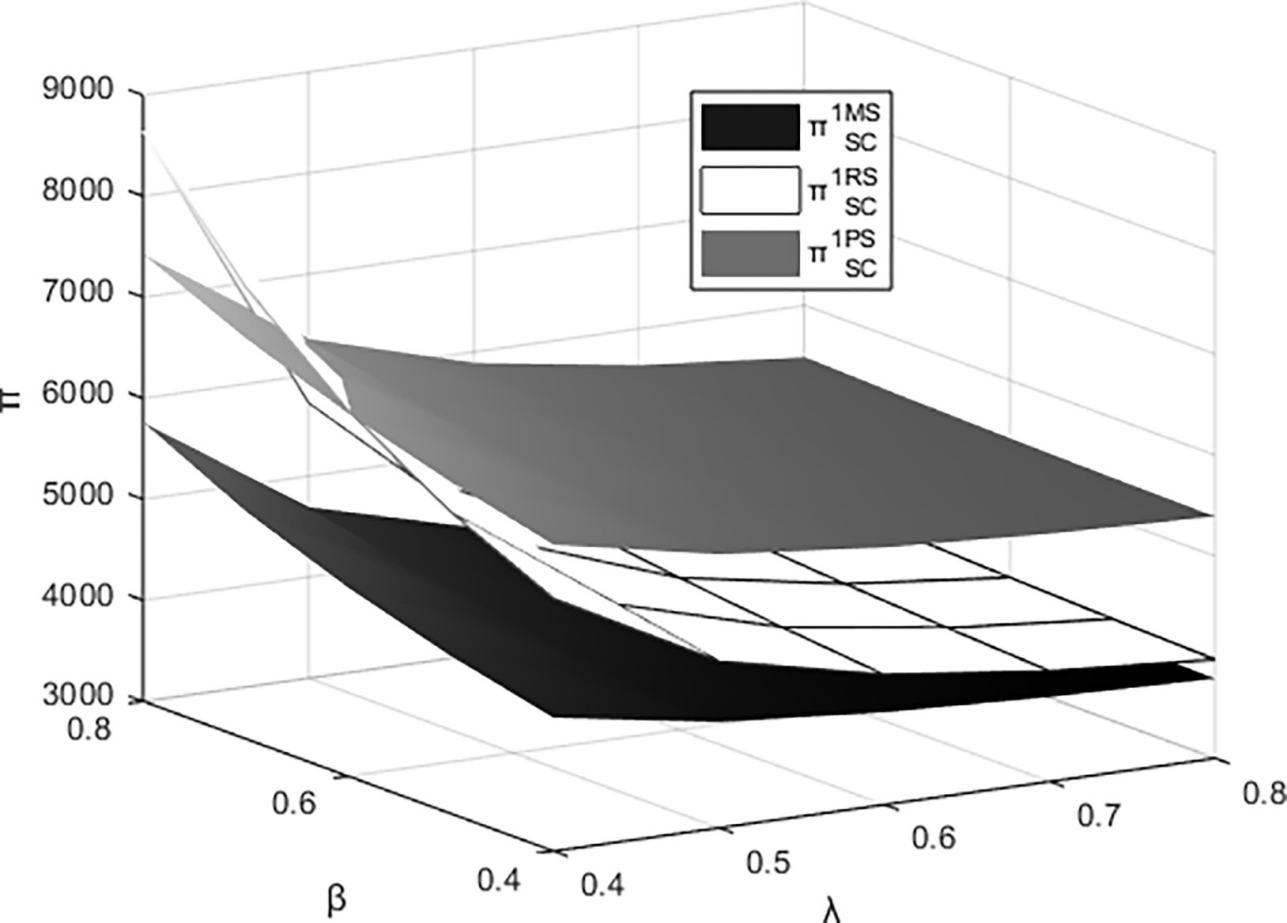
The impact of β and λ on the overall profitability of the platform supply chain.

Figs 11–14 respectively illustrate the joint impact of the events organizing committee’s low-carbon preference β and the platform technology investment’s carbon reduction impact coefficient λ on the individual profits of the materials supplier, the materials distributor, and the integrated service platform from their respective perspectives under different dominant structures. It can be observed from the figures that each entity attains maximum profits under the platform supply chain structure dominated by itself. The profits of the materials supplier and the materials distributor are most influenced by the events organizing committee’s low-carbon preference and the carbon reduction impact coefficient of platform technology investment when operating under their respective dominant supply chain structures. Meanwhile, the profits of integrated service platforms are most significantly influenced under the dominance of the materials distributor.

### 6.4. Profit variations among entities under a cost-sharing contract

**Fig 15:**
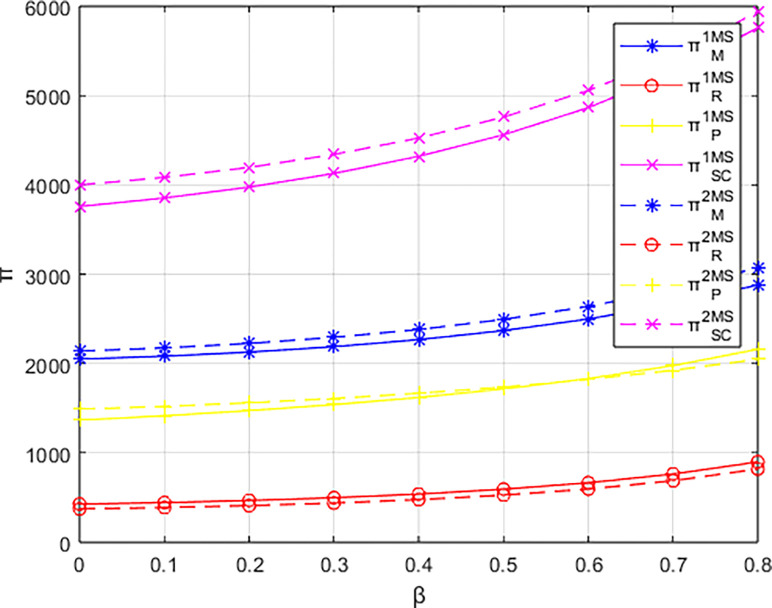
The profit comparison with and without contracts under the materials supplier-dominant structure.

**Fig 16:**
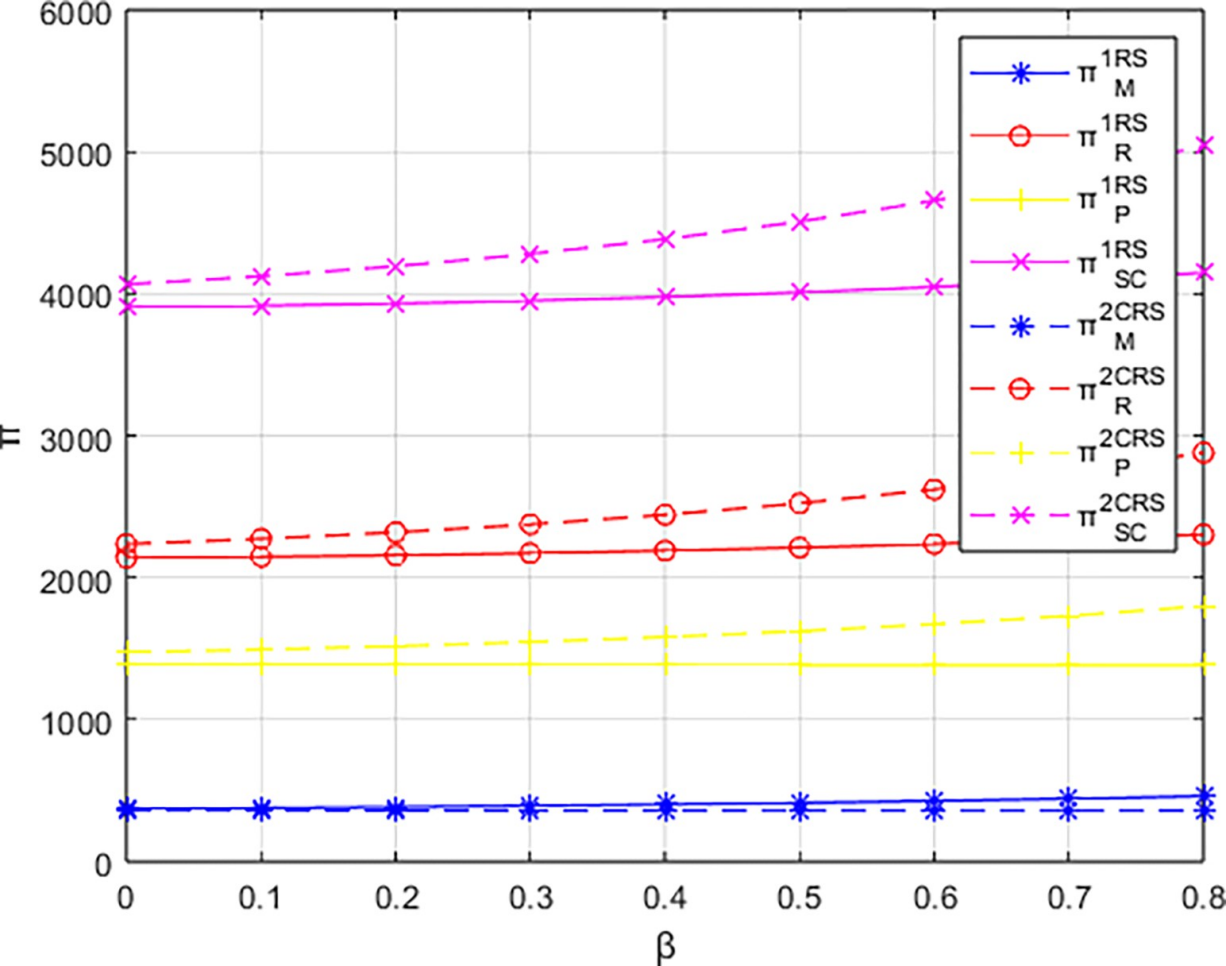
The profit comparison with and without contracts under the materials distributor-dominant structure.

**Fig 17:**
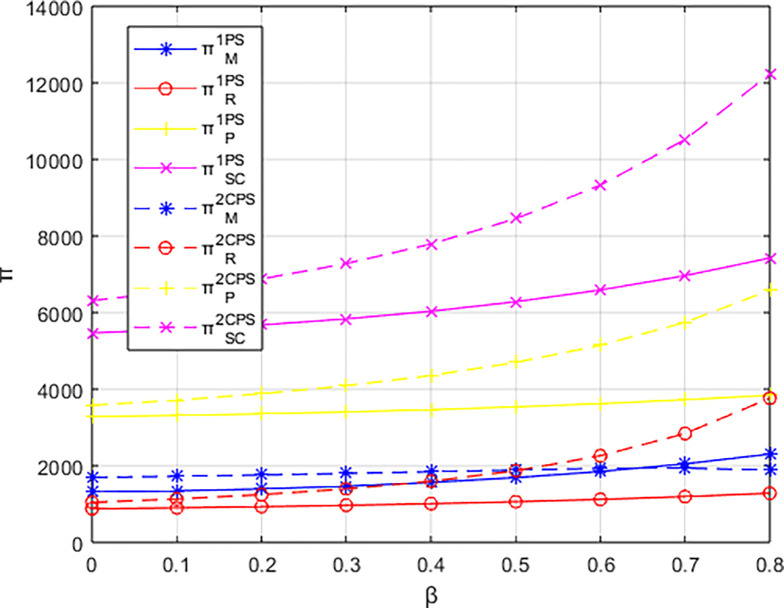
The profit comparison with and without contracts under the integrated service platform-dominant structure.

Conducting a baseline study on the impact of events organizing committee’s low-carbon preferences on the profitability of various entities, this research explores the effectiveness of cost-sharing contracts. Figs 15–17 compare the profits of various entities before and after implementing different cost-sharing contracts under different dominant structures.

Firstly, comparing the scenarios with and without a cost-sharing contract based on the profits of various parties: in the supplier-dominated structure, the overall profit of the platform supply chain and the supplier’s profit are both higher under the cost-sharing contract compared to the scenario without such a contract. The integrated service platform also shows a certain range of profit increase under the cost-sharing contract; however, the profit of the materials distributor is lower with the cost-sharing contract than without it. In the distributor-dominated structure, the overall profit of the platform supply chain, as well as the profits of the distributor and the integrated service platform, are higher under the cost-sharing contract compared to the corresponding profits without the contract. Conversely, the profit of the materials supplier decreases with the cost-sharing contract compared to the no-contract scenario. In the integrated service platform-dominated structure, the overall profit of the platform supply chain and the profits of all parties involved are higher under the cost-sharing contract compared to the no-cost-sharing scenario. However, the profit of the materials supplier, under the cost-sharing contract, tends to be lower than without the contract as the low-carbon preference of the event organizers increases.

Examining the magnitude of changes in profits with and without a contract, the implementation of a cost-sharing contract under the integrated service platform-dominated structure yields the largest increases in overall platform supply chain profit, distributor profit, and the profit of the integrated service platform itself. In contrast, under a supplier-dominated structure, the changes in profits with the cost-sharing contract are minimal, with the distributor-dominated structure showing intermediate results. Notably, under the distributor-dominated structure, the introduction of a cost-sharing contract shifts the relationship between the integrated service platform’s profit and the event organizers’ low-carbon preferences to a positive one. This means that, contrary to the no-contract scenario, the profit of the integrated service platform increases with the degree of low-carbon preference of the event organizers.

Overall, cost-sharing contracts increase the overall profit of the platform supply chain regardless of the dominant structure. However, the effectiveness of cost-sharing contracts is superior when the integrated service platform dominates compared to scenarios where the materials supplier or the distributor dominate.

## 7. Conclusions

The issue of carbon emissions in large-scale sports events is a critical topic based on the global pursuit of sustainable development. Achieving full-chain management of sports events materials from a supply chain perspective can facilitate carbon footprint management and risk management. By exploring each supply chain stage, it is possible to comprehensively reveal the potential of low-carbon pathways to enhance the sustainability and resilience of events operations. Additionally, resource management and partner collaboration at each stage can improve supply chain transparency and reduce the risk of disruptions within the supply chain. The construction of infrastructure for large-scale sports events is a major source of carbon emissions. We consider the role of the integrated service platform in providing low-carbon technology support within the infrastructure supply chain, innovatively incorporating the platform into the decision-making framework and exploring low-carbon cooperation coordination strategies under various power structures in the platform supply chain for large-scale sports events. This approach not only aligns with current trends in the development of supply chain and sports events within the digital economy but also significantly enriches the traditional research on supply chain power structures and the sustainable development of sports events. The findings provide decision-making support for the selection of partners and strategies during the self-organizing process of the various entities involved in large-scale sports events.

The main research conclusions and practical implications are as follows:

1. Under centralized decision-making, the profitability of the platform supply chain surpasses that under decentralized decision-making. This indicates that regardless of low-carbon collaboration, centralized decision-making is more conducive to achieving coordination among sports events materials platform supply chain. Centralized decision-making, compared to decentralized decision-making, facilitates more effective resource integration, unification of strategies, and enhances the overall resilience of the supply chain, ensuring controlled risk propagation. This centralized decision-making model is better suited to adapt to rapidly changing market environments, providing stronger support for the sustainable development of the supply chain. Therefore, when organizing sports events, the government can implement policies and measures to encourage all entities within the platform supply chain to work towards a unified strategic goal and enhance coordination and collaboration among them. For instance, the government could enforce control over sports events materials resources and establish a unified resource management system to coordinate the logistics, services, and facilities required for the events, thereby ensuring the efficient utilization of resources.
2. Cooperative innovation in the supply chain of the sports events materials platform can effectively enhance the overall efficiency, providing insights for low-carbon collaboration among the platform’s supply chain entities. In both collaboration scenarios, the dominant entities enjoy profit distribution advantages, with the integrated service platform having a stronger advantage. This dominance may lead to intense competition among entities, exacerbating supply chain risks and disrupting stability. To prevents excessive competition, dominant entities should use their power to regulate the competitive behavior of other supply chain entities, while also leveraging external means to ensure fair competition among the platform’s supply chain entities. For example, establishing clear industry standards and codes of conduct can guide other supply chain participants to operate within a fair competitive environment. Additionally, leveraging regulatory mechanisms such as those provided by the government and events organizing committees can ensure that competitive behaviors among participating entities are more reasonable and standardized. Furthermore, the events organizing committee, as the core of the event’s organization, can play a regulatory role by reviewing and providing feedback on supply chain participants, thereby promoting fair competition among the entities involved.
3. The carbon preference level of events organizing committees correlates positively with the overall profit of the sports events materials platform supply chain, the profit of the materials supplier, and the profit of the distributor. However, its relationship with the profit of the integrated service platform depends on the power structure within the supply chain. The impact coefficient of platform technology carbon reduction has the opposite effect on decisions. Therefore, each entity in the platform supply chain should carefully analyze the power structure when formulating low-carbon strategies. Emphasizing low-carbon and environmental considerations can be a beneficial strategy if the distributor and materials supplier have greater influence. However, the integrated service platform may require more flexible strategies to adapt to changes in power structures. Additionally, since the impact of events organizing committee’s carbon preferences and the platform technology carbon reduction coefficient on the profits of various platform supply chain entities is opposite, entities need to balance technological innovation and environmental goals. When adopting new technologies, it is essential to balance environmental objectives and economic benefits, ensuring that the application of technology does not significantly reduce the overall profit of the supply chain.
4. Under the dominance of the materials distributor, the carbon reduction level of products is highest, while under the dominance of the integrated service platform, the overall profit of the platform supply chain is maximized. Introducing cost-sharing contracts effectively enhances the overall profit of the sports events materials platform supply chain and the profits of individual entities, especially with a more favorable impact under the dominance of the integrated service platform. Different power structures within the platform supply chain leads to variations in overall objectives. The organizing committee with a preference for low-carbon tend to favor structures dominated by the carbon-efficient materials distributor, while those prioritizing financial interests may opt for materials supply chains dominated by the integrated service platform. Given the impact of the platform supply chain structure on strategic goals, it is advisable to establish a flexible sports events materials platform supply chain system, enabling entities to adapt partner relationships and low-carbon cooperation strategies accordingly.

The above conclusions and practical insights provide crucial scientific evidence for implementing low-carbon strategies in large-scale sports events. With the 2026 FIFA World Cup approaching, both FIFA and the global sports community are placing significant emphasis on the sustainability and environmental impact of the events to align with carbon neutrality commitments. As the organizing committee for the World Cup, FIFA should collaborate with the host government and effectively utilize its regulatory role to actively promote low-carbon cooperation among the entities within the events’ materials supply chain. Considering the impact of different supply chain power structures, FIFA should carefully select its platform supply chain partners and models. In response to the demand for low-carbon events, FIFA might opt for a supply chain model where the materials distributor hold absolute bargaining power or strengthen connections with the integrated service platform to ensure positive contributions to both the environment and the economy.

The limitations and future research directions of this study are primarily as follows: First, we construct only a linear supply chain structure and do not fully account for the unique network characteristics of platform supply chain in any of the models. The use of a single linear structure in our research may limit the generalizability of the conclusions. Future research could consider developing multi-tiered platform supply chain models and employing multi-level network game theory methods to further explore how low-carbon cooperation and innovation mechanisms operate within complex network environments. Moreover, differences among suppliers in terms of technology absorption and innovation capabilities may significantly impact their performance in low-carbon cooperation. Therefore, future research should also focus on the suppliers’ ability to absorb low-carbon innovation technologies, examining both internal and external factors involved in the implementation process. This includes aspects such as resource acquisition, capability development, and the effectiveness of technology investments. In summary, future studies should not only expand the structural models of supply chain but also emphasize the unique roles of other participants in the low-carbon innovation process to comprehensively advance the application and dissemination of low-carbon technologies.

## Supporting information

S1 Appendix Proof of Corollary 2. (DOCX)

S2 Appendix Proof of Corollary 3. (DOCX)

S3 Appendix Proof of Corollary 4 and Corollary 5. (DOCX)

## References

[1] Trendafilova S, McCullough B, Pfahl M; Nguyen SN, Casper J, Picariello M. Environmental sustainability in sport: Current state and future trends. Global Journal of Pure and Applied Sciences.2014; 3:9–14.

[2] McCullough BP, Pfahl ME, Nguyen SN. The green waves of environmental sustainability in sport. Sport in Society.2016; 19:1040–1065. doi: 10.1080/17430437.2015.1096251

[3] United Nations Climate Change Secretariat. Sports of climate action framework. UNFCC, Germany, 07 December 2018.

[4] Stephen E, Chalkley B. Mega-sporting events in urban and regional policy: A history of the Winter Olympics. Planning Perspectives.2004; 19:201–204. doi: 1080/0266543042000192475

[5] Ventura LMB, Ramos MB, Gioda A, França BB, de Oliveira Godoy JM. Air quality monitoring assessment during the 2016 Olympic Games in Rio de Janeiro, Brazil. Environmental Monitoring and Assessment 2019; 191:1–14. doi: 10.1007/s10661-019-7496-y 31093831

[6] Zhao J, Yin Z, Zhou G. The Strategic Analysis of Demand Forecast-Sharing in a Hybrid-Format Online Platform Supply Chain. Journal of Systems Science and Systems Engineering,2024; 33:3281–310. doi: 10.1007/s11518-024-5596-x

[7] Lu L, Mozart BM. Supply chain vertical competition and product proliferation under different power structures. International Journal of Production Economics.2024; 267:109097. doi: 10.1016/j.ijpe.2023.109097

[8] Liu T, Feng Q. R&D mode and coordination of green products in sustainable supply chain considering power structures. PLoS ONE. 2023; 18(11): e0291351. doi: 10.1371/journal.pone.0291351 37917625 PMC10621872

[9] Zhao L, Zhao Y. Impact of uncertain demand and channel power on dual channel supply chain decisions. PLoS ONE. 2024; 19(3): e0300386. doi: 10.1371/journal.pone.0300386 38489340 PMC10942094

[10] Chen N, Cai J, Kannan G. Decision analysis of supply chain considering yield uncertainty and CSR under different market power structures. Journal of Cleaner Production.2024; 434:139006. doi: 10.1016/j.jclepro.2023.139006

[11] Cheng Z, Zhou X, Zhou B, Zhao Z. Impacts of a mega sporting events on local carbon emissions: A case of the 2014 Nanjing Youth Olympics. China Economic Review.2022; 73:101782. doi: 10.1016/j.chieco.2022.101782

[12] Wang M, Bao Helen XH. Mega-events effects on the housing market: Evidence from the Beijing 2008 Olympic Games. Cities.2018; 72:207–216. doi: 10.1016/j.cities.2017.07.014

[13] Santos JO, Santos M. The right to housing, the world cup and the Olympics: Reflections on the case of Rio de Janeiro. Territorio.2013; 64: 28–33.

[14] Popelářová S, Janiga M. Winter Olympic Games as environmental problem. Oecologia Montana.2008; 17(1–2):34–40. https://om.vuvb.uniza.sk/index.php/OM/article/view/206.

[15] Chappelet JL. Olympic environmental concerns as a legacy of the Winter Games. International Journal of the History of Sport.2008; 25(14):1884–1902. doi: 10.1080/09523360802438991

[16] Khan SAR, Ponce P, Yu Z. Technological Innovation and Environmental Taxes toward a Carbon-free Economy: An Empirical Study in the Context of COP-21. Environmental Management.2021; 298:113418. doi: 10.1016/j.jenvman.2021.113418 34426217

[17] Ma T, Takeuchi K. Cleaning up the air for the 2008 Beijing Olympic Games: Empirical study on China’s thermal power sector. Resource and Energy Economics.2020; 60:101151. doi: 10.1016/j.reseneeco.2020.101151

[18] Zhang J, Zhong C, Yi M. Did Olympic Games improve air quality in Beijing? Based on the synthetic control method. Environmental Economics and Policy Studies.2016; 18:21–39. doi: 10.1007/s10018-015-0109-2

[19] Zhang C, Wang Q, Shi D, Li P, Cai W. Scenario-based potential effects of carbon trading in China: An integrated approach. Applied Energy.2016; 182:177–190. doi: 10.1016/j.apenergy.2016.08.133

[20] Zhao H, Zheng Y, Li T. Air quality and control measures evaluation during the 2014 Youth Olympic Games in Nanjing and its surrounding cities. Atmospherel.2017; 8(100): 177–190. doi: 10.3390/atmos8060100

[21] Ioannis S, Murat K, Timothy CB, Aza E, Hanin H., Bodour AM, et.al. How FIFA World Cup 2022? can meet the carbon neutral commitments and the United Nations 2030 Agenda for Sustainable Development?: Reflections from the tree nursery project in Qatar. Sustainable Development.2022; 30:203–226. doi: 10.1002/sd.2239

[22] Zhang Y. Artificial intelligence carbon neutrality strategy in sports events management based on STIRPAT-GRU and transfer learning. Frontiers in Ecology and Evolution.2023; 11. doi: 10.3389/fevo.2023.1275703

[23] BSI, Carbon Trust, DEFRA. PAS 2050:2008 specification for the assessment of the life cycle greenhouse gas emissions of goods and services. BSI, London, 29 October 2008.

[24] World Business Council for Sustainable Development & World Resources Institute. Product life cycle accounting and reporting standard. Wbcsd, Washington, DC, 22 September 2011.

[25] Matt D, Paul T. Reducing the carbon footprint of spectator and team travel at the University of British Columbia’s varsity sports events. Sport Management Review.2015; 18:244–255. doi: 10.1016/j.smr.2014.06.003

[26] Hana YAS, Tadesse W, Adeeb AK, Sehrish H, Muna A, Bajeela A, et al. How circular economy can reduce Scope 3 carbon footprints: Lessons learned from FIFA World Cup Qatar 2022.Circular Economy.2023; 2:100026. doi: 10.1016/j.cec.2023.100026

[27] Cooper JA, McCullough BP. Bracketing sustainability: Carbon footprinting March Madness to rethink sustainable tourism approaches and measurements. Journal of Cleaner Production.2021; 318:128475. doi: 10.1016/j.jclepro.2021.128475

[28] Fermeglia M. The Show Must Be Green: Hosting Mega-Sporting Events in the Climate Change Context. Carbon & Climate Law Review.2017; 11:100–109. https://www.jstor.org/stable/26353858.

[29] Gold S, Seuring S, Beske P. Sustainable supply chain management and inter-organizational resources: a literature review. Corporate Social Responsibility and Environmental Management.2010; 17:230–245. doi: 10.1002/csr.207

[30] Lu L, Hu S, Ren Y, Kang K, Li B. Research on Extension Design of Emergency Cold Chain Logistics from the Perspective of Carbon Constraints. Sustainability.2022; 14:1–22. doi: 10.3390/su14159083

[31] Ding H, Zhao Q, An Z, Tang O. Collaborative mechanism of a sustainable supply chain with environmental constraints and carbon caps. International Journal of Production Economics.2016; 181:191–207. doi: 10.1016/j.ijpe.2016.03.004

[32] Shaw K, Irfan M., Shankar R, Yadav SS. Low carbon chance constrained supply chain network design problem: a Benders decomposition based approach. Computers & Industrial Engineering.2016; 98:483–497. doi: 10.1016/j.cie.2016.06.011

[33] Cheng P, Wang T. Optimizing the emission control policies and trade-in program effects: A carbon-constrained closed-loop supply chain network model. Transportation Research Part E-Logistics and Transportation Review.2023; 179:103311. doi: 10.1016/j.tre.2023.103311

[34] Liu L, Li F. Differential game modelling of joint carbon reduction strategy and contract coordination based on low-carbon reference of consumers. Journal of Cleaner Production.2020; 277:123798. doi: 10.1016/j.jclepro.2020.123798

[35] Yu B, Wang J, Lu X, Yang H. Collaboration in a low-carbon supply chain with reference emission and cost learning effects: Cost sharing versus revenue sharing strategies. Journal of Cleaner Production.2020; 250:119460. doi: 10.1016/j.jclepro.2019.119460

[36] Zhou Y, Bao MJ, Chen X, Xu X. Co-op advertising and emission reduction cost sharing contracts and coordination in low-carbon supply chain based on fairness concerns. Journal of Cleaner Production.2016; 133:402–413. doi: 10.1016/j.jclepro.2016.05.097

[37] Zhao D, Xu C, Wang Q. Differential strategies of joint emission reductions and low-carbon promotion considering competing retailers. Journal of Control and Decision.2014; 29:1809–1815. doi: 10.13195/j.kzyjc.2013.1000

[38] Ghosh D, Shah J. Supply chain analysis under green sensitive consumer demand and cost sharing contract. International Journal of Production Economics.2015; 164:319–329. doi: 10.1016/j.ijpe.2014.11.005

[39] Yi Y, Li J. Cost-sharing contracts for energy saving and emissions reduction of a supply chain under the conditions of government subsidies and a carbon tax. Sustainability.2018; 10:895. doi: 10.3390/su10030895

[40] Drake DF, Kleindorfer PR, Van WLN. Technology Choice and Capacity Portfolios under Emissions Regulation. Production and Operations Management.2016; 25:1006–1025. doi: 10.1111/poms.12523

[41] Yu W, Wang Y, Feng W, Han R. Low carbon strategy analysis with two competing supply chain considering carbon taxation. Computers & Industrial Engineering.2022; 169:108203. doi: 10.1016/j.cie.2022.108203

[42] Sinayi M., Rasti-Barzoki M. A game theoretic approach for pricing, greening, and social welfare policies in a supply chain with government intervention. Journal of Cleaner Production.2018; 196:1443–1458. doi: 10.1016/j.jclepro.2018.05.212

[43] Cao K, Xu X, Wu Q, Zhang Q. Optimal production and carbon emission reduction level under cap-and-trade and low carbon subsidy policies. Journal of Cleaner Production.2017; 167:505–513. doi: 10.1016/j.jclepro.2017.07.251

[44] Saberi S, Cruz JM, Sarkis J, Nagurney A. A competitive multiperiod supply chain network model with freight carriers and green technology investment option. European Journal of Operational Research.2018; 266:934–949. doi: 10.1016/j.ejor.2017.10.043

[45] Wu X, Li S. Impacts of CSR Undertaking Modes on Technological Innovation and Carbon-Emission-Reduction Decisions of Supply Chain. Sustainability.2022; 14:13333. doi: 10.3390/su142013333

[46] Liu Z, Huang Y, Shang W, Zhao Y, Yang Z, Zhao Z. Precooling energy and carbon emission reduction technology investment model in a fresh food cold chain based on a differential game. Applied Energy.2022; 326:119945. doi: 10.1016/j.apenergy.2022.119945

[47] Chi JL, Chih TY, Hsiu FY. Stackelberg game approach for sustainable production-inventory model with collaborative investment in technology for reducing carbon emissions. Journal of Cleaner Production.2020; 270:121963. doi: 10.1016/j.jclepro.2020.121963

[48] Pan H, Zhu H, Teng M. Low-Carbon Transformation Strategy for Blockchain-Based Power Supply Chain. Sustainability.2023; 15:12473. doi: 10.3390/su151612473

[49] Jiang W, Shi K, Zhang L, Jiang W. Modelling of pricing, crashing, and coordination strategies of prefabricated construction supply Chain with power structure. PLoS ONE.2023; 18(8): e0289630. doi: 10.1371/journal.pone.0289630 37561742 PMC10414617

[50] Liu J, Tian Q, Zhang N. Research the effect of anticipated regret and fairness concerns on retailer-led supply chain. PLoS ONE.2023; 18(1): e0279334. doi: 10.1371/journal.pone.0279334 36652490 PMC9847980

[51] Xia L, Gu M, Li D, Wang J. Pricing strategies of supply chain with asymmetric demand information considering equity holding under different power structures. Chinese Journal of Managenent Science.2024; 1–16. doi: 10.16381/j.cnki.issn1003-207x.2023.0709

[52] Niu W, Shen H, Zhang L. Impact of power structure on decarbonizing investment with uncertain innovation: A triple bottom line perspective. Transportation Research Part E-Logistics and Transportation Review.2022; 160:102654.

[53] Huang D, Zhang J. The impacts of carbon tax on emissions abatement level in a supply chain under different power structures. Chinese Journal of Management Science.2021; 29:57–70. doi: 10.16381/j.cnki.issn1003-207x.2018.1761

[54] Fan D, Xu Q. Decisions of enterprises carbon emission reduction and government subsidy in different power structures. Soft Science. 2018; 32: 64–70. doi: 10.13956/j.ss.1001-8409.2018.12.14

[55] Chot TM, Li Y, Xu L. Channel leadership, performance and coordination in closed loop supply chains. International Journal of Production Economics.2013; 146:371–380. doi: 10.1016/j.ijpe.2013.08.002

[56] Kou X, Liu H, Gao H, Liu H, Yu H. Cooperative emission reduction in the supply chain: the value of green marketing under different power structures. Environmental Science and Pollution Research. 2022; 29:68396–68409. doi: 10.1007/s11356-022-20683-3 35543782

[57] Gong W, Xia M, Ding F, Sun L. Dynamic decisions of dual-channel green supply chain considering power structures under manufacturer’s overconfidence. Computer Integrated Manufacturing Systems.2024; 1–17. doi: 10.13196/j.cims.2023.0480

[58] Luo X, Wang Z, Lu L, Guan Y. Supply Chain Flexibility Evaluation Based on Matter-Element Extension. Complexity.2020; 5:1–12. doi: 10.1155/2020/8057924

[59] Lu L, Su X, Hu S, Luo X, Liao Z, Ren Y, et al. Green transition in manufacturing: Dynamics and simulation. PLoS ONE.2023; 18:1–26. doi: 10.1371/journal.pone.0280389 36662689 PMC9858057

