# Supplementary material for: Coordination strategy and contract design of platform supply chain for large-scale sports events with low carbon preference
Source: PLoS One. 2024 Dec 2;19(12):e0311086. doi: 10.1371/journal.pone.0311086 (PMC11611220; doi:10.1371/journal.pone.0311086)
Supplement: S1 Appendix — (DOCX) [file pone.0311086.s001.docx]

Supporting information 1

S1 Appendix. Proof of Corollary [2](#corollary2)

${\pi_{SC}}^{0}-{\pi_{SC}}^{1}=\frac{\left[ -C\left( t\alpha+\beta\right)^{2}+4\alpha^{2}\lambda^{2}{u_{p}}^{2}\left( \alpha-1 \right) \right]B^{2}}{4\alpha C^{2}}$ , as $c>0$and $0<\alpha<1$, it follows that $\left[ -C\left( t\alpha+\beta\right)^{2}+4\alpha^{2}\lambda^{2}{u_{p}}^{2}\left( \alpha-1 \right) \right]<0$, therefore ${\pi_{SC}}^{0}-{\pi_{SC}}^{1}<0$.

${\pi^{1}}_{M}^{MS}-{\pi^{0}}_{M}^{MS}=\frac{\beta\left( t\alpha+\beta\right)B^{2}}{24\alpha\left( -\beta\left( t\alpha+\beta\right)+3\alpha\lambda^{2}u_{p} \right)}$, as $3\alpha\lambda^{2}u_{p}-t\alpha\beta-\beta^{2}>0$, it follows that ${\pi^{1}}_{M}^{MS}-{\pi^{0}}_{M}^{MS}>0$.

${\pi^{1}}_{R}^{MS}-{\pi^{0}}_{R}^{MS}$*=*$\frac{B^{2}\left[ -\beta^{2}\left( t\alpha+\beta\right)^{2}+6\alpha\beta\left( t\alpha+\beta\right)\lambda^{2}u_{p}-9\left( -1+\alpha\right)\alpha\lambda^{4}u_{p}^{2} \right]}{144\alpha\left( \beta\left( t\alpha+\beta\right)-3\alpha\lambda^{2}u_{p} \right)^{2}}$.Given $A=2\alpha\lambda^{2}u_{p}-\beta^{2}>0$ and ${C=2\alpha\lambda^{2}u_{p}-\left( t\alpha+\beta\right)}^{2}>0$, it follows that $\beta^{2}<\left( t\alpha+\beta\right)^{2}<2\alpha\lambda^{2}u_{p}$. Therefore $-\beta^{2}\left( t\alpha+\beta\right)^{2}+6\alpha\beta\left( t\alpha+\beta\right)\lambda^{2}u_{p}-9\left( -1+\alpha\right)\alpha\lambda^{4}u_{p}^{2}>-\beta^{2}\left( t\alpha+\beta\right)^{2}+6\alpha\beta^{2}\lambda^{2}u_{p}+9\left( 1-\alpha\right)\alpha\lambda^{4}u_{p}^{2}=\beta^{2}\left[ 6\alpha\lambda^{2}u_{p}-\left( t\alpha+\beta\right)^{2} \right]+9\left( 1-\alpha\right)\alpha\lambda^{4}u_{p}^{2}>0$. Consequently, ${\pi^{1}}_{R}^{MS}-{\pi^{0}}_{R}^{MS}>0$.

${\pi^{1}}_{P}^{MS}-{\pi^{0}}_{P}^{MS}=\frac{B^{2}\beta[-2\beta\left( t\alpha+\beta\right)^{2}+3\alpha\lambda^{2}u_{p}(4t\alpha+\beta))]}{72\alpha\left( \beta\left( t\alpha+\beta\right)-3\alpha\lambda^{2}u_{p} \right)^{2}}$. The sign of the expression $-2\beta\left( t\alpha+\beta\right)^{2}+3\alpha\lambda^{2}u_{p}\left( 4t\alpha+\beta\right)$ determines whether it is positive or negative. Therefore, ${\pi^{1}}_{P}^{MS}-{\pi^{0}}_{P}^{MS}<0$ when $-2\beta\left( t\alpha+\beta\right)^{2}+3\alpha\lambda^{2}u_{p}\left( 4t\alpha+\beta\right)<0$. Given that $\beta^{2}<\left( t\alpha+\beta\right)^{2}<2\alpha\lambda^{2}u_{p}$ ,it can be inferred that $3t\alpha-\beta<0$. Thus, when $3t\alpha-\beta>0$, ${\pi^{1}}_{P}^{MS}-{\pi^{0}}_{P}^{MS}>0$.

Similarly, the other conclusions can be proven.
