# Supplementary material for: Coordination strategy and contract design of platform supply chain for large-scale sports events with low carbon preference
Source: PLoS One. 2024 Dec 2;19(12):e0311086. doi: 10.1371/journal.pone.0311086 (PMC11611220; doi:10.1371/journal.pone.0311086)
Supplement: S2 Appendix — (DOCX) [file pone.0311086.s002.docx]

Supporting information 2

S2 Appendix. Proof of Corollary [3](#corollary3)

${\pi^{1}}_{R}^{MS}-{\pi^{1}}_{M}^{MS}=\frac{\lambda^{2}Bu_{p}\left[ 2\beta\left( t\alpha+\beta\right)+\left( 1-6\alpha\right)\lambda^{2}u_{p} \right]}{16\left[ \beta\left( t\alpha+\beta\right)-3\alpha\lambda^{2}u_{p} \right]^{2}}$,let$2\beta\left( t\alpha+\beta\right)+\left( 1-6\alpha\right)\lambda^{2}u_{p}=\left( t\alpha+\beta\right)^{2}-t^{2}r^{2}+\beta^{2}-6\alpha\lambda^{2}u_{p}$+ $\lambda^{2}u_{p}$，because $0<\alpha<1$, then $\lambda^{2}u_{p}< \alpha\lambda^{2}u_{p}$,which implies $2\beta\left( t\alpha+\beta\right)+\left( 1-6\alpha\right)\lambda^{2}u_{p}<\left( t\alpha+\beta\right)^{2}-t^{2}r^{2}+\beta^{2}-5\alpha\lambda^{2}u_{p}$. Also, since$\beta^{2}<{(t\alpha+\beta)}^{2}<2\alpha\lambda^{2}u_{p}$,it follows that $2\beta\left( t\alpha+\beta\right)+\left( 1-6\alpha\right)\lambda^{2}u_{p}<{(t\alpha+\beta)}^{2}-t^{2}r^{2}+\beta^{2}-5\alpha\lambda^{2}u_{p}<-\alpha\lambda^{2}u_{p}-t^{2}r^{2}<0$. Therefore ${\pi^{1}}_{R}^{MS}-{\pi^{1}}_{M}^{MS}<0$.

${\pi^{1}}_{P}^{MS}-{\pi^{1}}_{M}^{MS}=\frac{\alpha\lambda^{2}B^{2}u_{p}\left( t\beta-\lambda^{2}u_{p} \right)}{8\left( \beta\left( t\alpha+\beta\right)-3\alpha\lambda^{2}u_{p} \right)^{2}}$,because $\beta^{2}<{(t\alpha+\beta)}^{2}<2\alpha\lambda^{2}u_{p}，t\beta-\lambda^{2}u_{p}$=$\frac{2t\alpha\beta-{2\alpha\lambda}^{2}u_{p}}{2\alpha}$=$\frac{{(t\alpha+\beta)}^{2}-{2\alpha\lambda}^{2}u_{p}-\beta^{2}-t^{2}\alpha^{2}}{2\alpha}<0$,therefore ${\pi^{1}}_{P}^{MS}-{\pi^{1}}_{M}^{MS}<0$.

The other conclusions can be deduced by analogous reasoning.
