# Supplementary material for: Coordination strategy and contract design of platform supply chain for large-scale sports events with low carbon preference
Source: PLoS One. 2024 Dec 2;19(12):e0311086. doi: 10.1371/journal.pone.0311086 (PMC11611220; doi:10.1371/journal.pone.0311086)
Supplement: S3 Appendix — (DOCX) [file pone.0311086.s003.docx]

Supporting information 3

S3 Appendix. Proof of Corollary [4](#corollary4) and Corollary [5](#corollary5)

${\pi^{1}}_{SC}^{PS}-{\pi^{1}}_{SC}^{RS}>\frac{\lambda^{2}B^{2}u_{p}[-4\left( t\alpha+\beta\right)\left( 5t\alpha+\beta-4\alpha\beta\right)+101\alpha\lambda^{2}u_{p}]}{16\left( \left( t\alpha+\beta\right)^{2}-8\alpha\lambda^{2}u_{p} \right)^{2}}>\frac{\lambda^{2}B^{2}u_{p}[30.5t\alpha+36.5\beta+16\alpha\beta]}{16\left( \left( t\alpha+\beta\right)^{2}-8\alpha\lambda^{2}u_{p} \right)^{2}}>0$; ${\pi^{1}}_{SC}^{RS}-{\pi^{1}}_{SC}^{MS}>\frac{t\alpha\lambda^{2}B^{2}u_{p}\left[ 2\left( t\alpha+\beta\right)^{2}t\alpha\right]}{16\left( \beta\left( t\alpha+\beta\right)-3\alpha\lambda^{2}u_{p} \right)^{2}C^{2}}>0$. ${e_{\triangle}}_{PS}^{1}-{e_{\triangle}}_{RS}^{1}<0$;${e_{\triangle}}_{MS}^{1}-{e_{\triangle}}_{RS}^{1}<0$;When ${e_{\triangle}}_{PS}^{1}-{e_{\triangle}}_{MS}^{1}<0$, ${e_{\triangle}}_{PS}^{1}-{e_{\triangle}}_{MS}^{1}<0$. The specific proof process is identical to the aforementioned; thus, redundant elaboration is omitted.
